# Microwave Radiation for Organolithium Chemistry: Mechanistic Studies on the Direct *α*‐Metalation of a Tertiary Amine

**DOI:** 10.1002/chem.202502149

**Published:** 2025-08-14

**Authors:** Annika Schmidt, Rebecca Scheel, Andrea Ost, Lukas Brieger, Carsten Strohmann

**Affiliations:** ^1^ Inorganic Chemistry, TU Dortmund University Otto‐Hahn‐Str. 6 44227 Dortmund Germany

**Keywords:** amines, butyllithium, deprotonation, microwave, organolithium

## Abstract

Deprotonation reactions with lithium alkyls exhibit a high temperature dependency. However, the reactivity of lithium alkyls with conventionally used Lewis bases limits their deprotonation capability of more challenging substrates like tertiary amines. Therefore, we have focused on the use of high but exactly set reaction temperatures by usage of microwave radiation in the deprotonation reaction of a tertiary amine in the absence of polar additives. Our study proves the robustness of lithium alkyls, like *t*‐butyllithium and *α*‐metalated tertiary amines under microwave conditions. A solid‐state structure of the prelithiation aggregate and quantum chemical calculations offer an insight into the mechanism, revealing that the substrate amine performs as Lewis base for deaggregation itself. By our method, *N*‐methylpiperidine was deprotonated without any intermediate or possibly toxic reaction steps, solvents, or additives, solely with *t*‐butyllithium in the microwave with 56% yield. By using *design of experiments* we identified the most important factors for a successful deprotonation, revealing the closed reaction conditions under pressure and an excess of amine to be crucial. This investigation proves the high potential of microwave radiation in lithium chemistry, as it provides high but exact energy and introduces new possibilities for deprotonation reactions to overcome high kinetic reaction barriers.

## Graphical abstract

We present the use of microwave radiation to lithium chemistry, specifically in the kinetically hindered deprotonation of a tertiary amine at high temperatures without polar additives. The amine acts as substrate and deaggregation agent as shown by a solid‐state structure. The lithium compounds remain stable at the harsh conditions and the deprotonated amine is storage‐stable, facilitating diverse follow‐up chemistry.

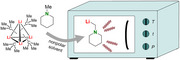

## 1 Introduction

**Scheme 1:**
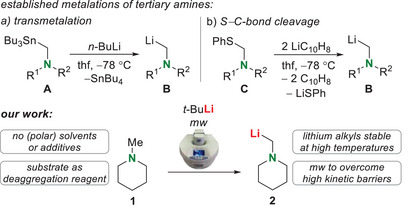
Known methods for the synthesis of *α*‐metalated tertiary amines,^[^ ^6^ ^]^ and the method of microwave‐based deprotonation presented in this work (R^1^, R^2^ = organic substituents).

Deprotonation reactions with lithium alkyls are a widely used synthetic method in organic and inorganic chemistry.^[^ ^1^ ^]^ However, the successful use of lithium alkyls as reagents is not trivial due to their close structure‐reactivity relationship and is directly related to the reaction conditions of temperature, solvent, and possible additives.^[^ ^2^ ^]^ The deaggregation of the lithium alkyl plays a major role to enhance the reactivity, hence many investigations focus on the use of polar and commercially available reagents.^[^ ^3^, ^4^ ^]^ However, this results in some limitations. Classical polar additives, such as tetrahydrofuran (thf), tetramethylethylenediamine (tmeda), or diethyl ether decompose in the presence of lithium alkyls at temperatures above –20 °C and thus only allow a defined temperature range for lithiations.^[^ ^5^ ^]^ Therefore, not every substrate is accessible for classical deprotonations with lithium alkyls. For example, the *α*‐metalation of tertiary amines needs to be carried out via transmetalation reactions or C─S‐bond cleavage reactions, which require various reaction steps and produce multiple byproducts (Scheme 1).^[^ ^6^ ^]^

For several years microwave technology has been used successfully in the field of organic synthesis and catalysis and has led to new and optimized possibilities for many reactions.^[^ ^7^ ^]^ The combination of high energetic radiation, shortened reaction times, and an uncomplicated reaction setup has already led to groundbreaking achievements in these fields. Compared to conventional heating, microwave radiation has a higher efficiency, better heat management, and more precise adjustment possibilities. However, for effective heating, the reagents or solvents must be able to interact with the microwave radiation. This can proceed via dipoles or ions.^[^ ^8^ ^]^

## 2 Results and Discussion

Herein, we present the application of microwave radiation for lithium alkyl chemistry on the example of *α*‐deprotonation of *N*‐methylpiperidine with *t‐*butyllithium (Scheme 1, bottom). By using high reaction temperatures and the targeted heat conduction and exact temperature setting of the microwave, the previous limitation of a high kinetic barrier can be overcome. The substrate itself was able to deaggregate the lithium alkyl as shown by X‐ray diffraction, so that an additional polar reagent can be dispensed, rendering the lithium alkyls stable under the applied conditions. DFT‐calculations and in situ FT‐IR studies gave first hints on an *α*‐deprotonation of tertiary amines at elevated temperatures and by a statistical *design of experiments*, the microwave technique and precise knowledge of the stoichiometry were shown to be crucial for a successful *α*‐deprotonation. The developed method uses easy and not laborious reaction steps and can be applied to versatile follow‐up chemistry.

First, the temperature stability of the compounds needed to be evaluated. As lithium alkyl we chose *t‐*butyllithium (**3**) due to its good availability and properties although the temperature lability of butyllithiums in apolar solvents has already been shown.^[^ ^9^ ^]^ However, with the good heat conduction possibilities and precise adjustment possibilities of the microwave its degradation was hoped to be suppressed.

**Scheme 2:**
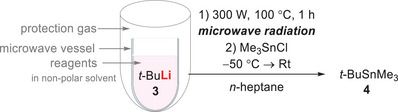
Preparation of the reaction solution for application under microwave radiation and reaction conditions for the usage of *tert*‐butyllithium in *n*‐heptane under high reaction temperatures.

In a first step, *t‐*butyllithium (**3**) was treated with microwave radiation to check whether it is stable under these conditions and to prove that no side reactions occur (Scheme 2). The attempt was carried out in Schlenk flasks, in which the microwave vessels could be evacuated, filled with inert gas and reagents (Figure S1). By using higher boiling *n*‐heptane instead of short‐chain *n*‐pentane or *n*‐hexane, it was possible to heat the mixture up to 100 °C with microwave radiation. Due to the highly symmetrical lithium alkyl oligomer (*t*‐butyllithium tetramer) in apolar solution,^[^ ^10^ ^]^ the heating took place slowly, which shows that only a small dipole moment is present that can interact with the microwave radiation. After heating the reaction solution for 1 hour and trapping the reaction mixture with trimethylchlorostannane, only the *t‐*butyl substituted compound **4** could be detected by GC/EI‐MS (see Supporting Information). The application of microwave radiation to butyllithium is therefore possible and the first fundamental question of lithium alkyl stability under microwave conditions is clarified.

As a preliminary example for the feasibility of deprotonation reactions with lithium alkyls under microwave conditions, we chose the direct *α*‐metalation of tertiary amines with the example substrate of *N*‐methylpiperidine. This reaction is not accessible by traditional means due to kinetic inhibition and is only feasible through laborious, atom‐inefficient reaction pathways (Scheme 1).^[^ ^6^ ^]^ Traditionally, the deaggregation of lithium alkyls is important for an efficient reaction control. This becomes even more important regarding their use under microwave radiation, since dipoles in the present structure are crucial for effective heating. For this reason, the ability of *N*‐methylpiperidine to deaggregate a lithium alkyl polyhedron was evaluated and investigated regarding possible reactive structural motifs.

**Figure 1:**
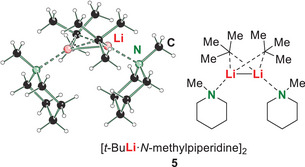
Solid‐state structure from X‐ray diffraction of dimeric *t*‐butyllithium with *N*‐methylpiperidine (**5**).

In this context, molecular structure **5** in the solid‐state was obtained by crystallization of *t*‐butyllithium (**3**) with *N*‐methylpiperidine (**1**) without prior treatment with microwave radiation (Figure 1). Compound **5** crystallizes in the orthorhombic crystal system in the space group *P*2_1_2_1_2_1_. The structure consists of a dimeric structural motif, which shows that *N*‐methylpiperidine can deaggregate the formerly tetrameric *t‐*butyllithium^[^ ^10^ ^]^ and break it down into smaller aggregates. In previous studies, dimeric motifs have already shown great potential for deprotonation reactions, since they form a compromise between a small aggregate and an accessible reactive center.^[^ ^11^ ^]^ Dimer **5** has also been characterized in solution via NMR (see S13–S15).

**Scheme 3:**
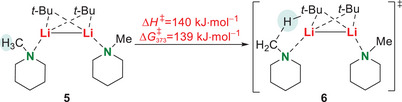
Schematic overview of quantum chemically calculated energy barrier between the dimeric compound **5** and the corresponding transition state **6** regarding the abstraction of a proton (B3LYP/6–31+g(d,p) *gd3*; *T* = 373 K).

In order to set the obtained structural motif of the deaggregated species in the context of the deprotonation reaction with the organolithium alkyl, we considered the energy barrier of the deprotonation step by quantum chemical calulations (Scheme 3). These were carried out at the B3LYP/6–31+g(d,p) *gd3‐*level of theory starting from the dimeric structure **5** toward the deprotonation of the respective amine. The abstraction of the proton can be realized with an energy barrier of 139 kJ·mol^−1^. This comparably high energy barrier is consistent with the experimental observation that no deprotonation takes place at ambient temperature.

**Scheme 4:**
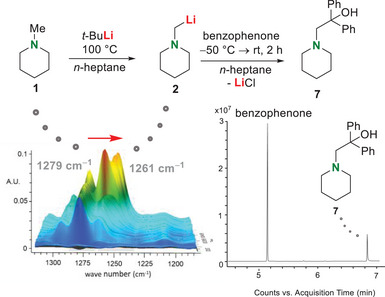
Deprotonation of *N‐*methylpiperidine (**1**) with *t*‐butyllithium under standard convection methods followed by in situ FT‐IR‐spectroscopy (top). Insight into the in situ FT‐IR‐spectroscopic surface and shift of the amine signal to 1261 cm^−1^ (left). Excerpt of the GC/EI‐MS spectrum after trapping with benzophenone (right).

To overcome these expected high barriers, the usage of higher reaction temperatures, and particularly the use of microwave radiation and its high adjustability was tested. The first experiment was carried out under conventional heating conditions. In situ FT‐IR‐spectroscopy was used to determine if the metalation is possible under standard convection methods (Scheme 4). *N‐*methylpiperidine (**1**) was treated with *t‐*butyllithium (**3**) in the nonpolar solvent *n*‐heptane and heated under conventional methods while IR spectra were constantly recorded.

The stepwise addition of the reagents enabled a clear assignment of the signals to the respective reactants (Scheme 4). Under conventional heating, a new IR frequency was obtained in the course of the reaction time, which resulted from the shift of a signal associated with the amine ($\tilde{\upsilon }$ = 1261 cm^−1^). After subsequent trapping with the electrophile benzophenone, a signal of the monosubstituted product **7** could be obtained by GC/EI‐MS and a yield of 5% was calculated from the ^1^H‐NMR spectrum (see Supporting Information, Figure S6).

Accordingly, the metalation of this substrate takes place under conventional heating. We tried microwave radiation as a heat source to make heating more efficient and to achieve a high, targeted energy input without heat gradients inside the flask. Thereby, we aimed to realize reproducibility at the border of the boiling point of **5** and keep amine **5** in the reaction solution throughout the reaction time. A prerequisite for the use of microwave radiation is the interaction of the reagents due to their dipole moments with the high energy microwave radiation. Quantum chemical calculations at the B3LYP/6–31+g(d,p) *gd3*‐level of theory reveal that *N*‐methylpiperidine (**1**) has a dipole moment of 0.5 Debye whereas the deaggregated dimeric species of *t‐*butyllithium with *N*‐methylpiperidine (**5**) has a much higher dipole moment of 4.34 Debye. Thus, it can be assumed, that the species formed in situ is able to interact effectively with the microwave radiation and thus ensures a homogeneous temperature distribution. This offers a short heating phase of a few minutes and allows a more precise setup of the reaction parameters.

**Scheme 5:**
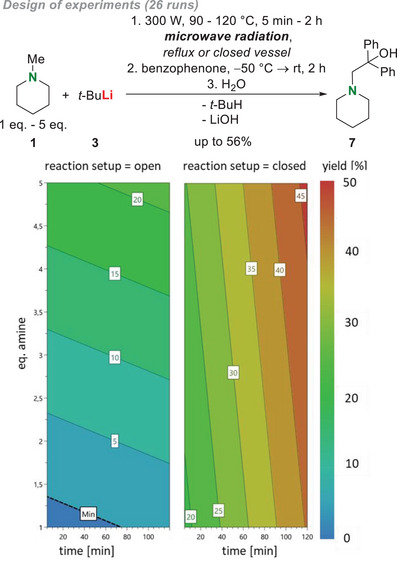
*Design of experiments* of deprotonation of *N‐*methylpiperidine (**1**) with *t*‐butyllithium (**3**) under microwave conditions. Time, temperature, equivalents of amine, and reaction setup were screened. Elevated times and a closed reaction setup were identified as the most important factors, while a higher amount of amine is also beneficial.^[^ ^12^ ^].^

Preparatively, there are two options for carrying out the sensitive reactions in the microwave. One route takes place in an open *Schlenk* flask in the microwave device fitted with a reflux condenser that allowed the reaction to reflux using microwave radiation. Another way is by heating in a closed microwave vessel, which was prepared and filled using a uniquely designed *Schlenk* flask, whereby reaction processes can also be carried out under pressure and allow a higher energy uptake. These two preparative possibilities for handling lead to a wide range of reaction control options for efficient heating and a controllable energy supply. To identify the most important factors for a successful *α*‐deprotonation, a statistical *design of experiments* (DoE) optimization was carried out. We concentrated on the factors time (5 minutes; 1 hour; 2 hours), temperatures below and above the boiling point of *N*‐methylpiperidine (90 °C; 100 °C; 120 °C), equivalents of amine (1 eq.; 2 eq.; 5 eq.) and reaction setup (open/reflux = 0; closed vessel = 1) with a quadratic process model for optimization of the reaction yield with 24 runs and 3 replicated runs (for more information, see Supporting Information). To further improve the usability of our method, we decided to not use any solvent, but conduct the *design of experiments* solvent‐free. After the reaction, benzophenone (in diethyl ether) was added to the solution at −50 °C to secure a homogeneous electrophilic trapping reaction and the analytical yield was calculated from ^1^H‐NMR. The results of this optimization can be seen in Scheme 5.

The NMR spectra proved the deprotonation at the methyl group of **1**. A consideration of the four different factors showed especially elevated time, high equivalents of amine, and a closed reaction setup to be beneficial for high yields. In the applied DoE model temperature did not play a role. The closed vessel that allows reactions under pressure proves that the microwave radiation is crucial for a successful *α*‐deprotonation of *N*‐methylpiperidine, which may be even more important for low boiling tertiary amines or different reaction systems with lithium organyls that rely on the temperature constancy or the controlled heat gradient of the microwave setup. As the reaction was carried out under microwave conditions, the pressure control in the microwave allows that the reaction can also be carried out in a closed system without any safety risks in comparison to heating in a closed vessel under standard convection methods (see Supporting Information, Figure S51). Also, an excess of the amine is needed, which is in accordance with the solid‐state structure **5**, the quantum chemical calculations and the consideration, that *N*‐methylpiperidine is substrate and deaggregation reagent at the same time. This further underlines the fact that no polar solvent or additive is needed in our presented method.

Here, we obtained a maximum yield of 56% (conditions: 100 °C, 120 minutes, 5 eq. amine, closed setup). In comparison to the traditional methods in Scheme 1, it should be emphasized that the reaction takes place in a few minutes within one reaction step with an easy to evaporate educt and without additional solvents or toxic intermediates.

**Scheme 6:**
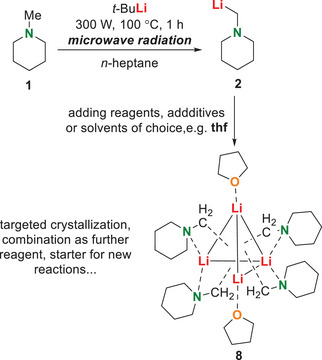
Application of the method and subsequent reaction with polar reagents led to targeted crystallization.

Another advantage of our microwave‐based deprotonation reaction is the flexibility regarding the further use of the lithiated component. Due to the synthesis without solvents, the lithiated compound shows a high stability and can thus also be stored in a nonpolar solvent under protective gas, similarly to commercially available lithium alkyls. The required polar additives, that are crucial for (de)aggregation of the lithium alkyl for subsequent applications, can be added at a later stage in the desired stoichiometry. As an example, metalated *N*‐methylpiperidine (**1**) can be specifically crylized as was already documented by steinborn et al.^[^ ^13^ ^]^ Here, this compound was synthesized by microwave synthesis, whereas steinborn et al. chose a transmetalation of the tributyltin derivative for synthesis (see Scheme 1a). After subsequent addition of thf and crystallization, compound **8** was obtained (Scheme 6).

## 3 Conclusions

To conclude, we have shown that microwave radiation offers an approach for kinetically hindered deprotonation reactions, presented by the example of *α*‐metalation of *N*‐methylpiperidine (**1**) with *t*‐butyllithium (**3**) under elevated reaction temperatures. Both, *t*‐butyllithium (**3**) and the *α*‐metalated amine **2** proved to be stable under the microwave conditions applied. Due to the absence of polar additives, the substrate amine itself can deaggregate the parental lithium alkyl aggregate as was shown by dimeric solid‐state structure **5**. Starting from this solid‐state structure, quantum chemical calculations were performed that confirm the expected high kinetic barrier. The usability of higher reaction temperatures was proved in a classical convection experiment followed by in situ FT‐IR spectroscopy. By *design of experiments*, the reaction yield was improved to 56% and the most important optimization factor was proven to be the closed vessel microwave setup. Due to the absence of (polar) solvents the lithiated amines are characterized by later storage stability and the possibility of adding versatile solvents in desired stoichiometric quantities for subsequent applications.

With this work, we show that the application of microwave radiation with its high and targeted energy input by setting exactly the desired temperature can be seen as an interesting tool to selectively overcome high kinetic deprotonation barriers. The stability of lithium alkyls under microwave conditions and high temperatures due to the absence of (polar) solvents or chelating ligands and the possibility for facile follow‐up chemistry render our method applicable in everyday laboratory work. Additionally, future systems may profit from the good temperature constancy of the microwave setup and the easy handling for reactions under pressure without safety risks. In future studies we will explore the potential of amines as ligands for the lithium alkyl mediated metalation of further kinetically hindered substrates like hydrocarbons under high reaction temperatures with microwave radiation.

## 4 Experimental Section

Experimental details of the lithium alkyl stability experiment, the in situ FTIR experiment under conventional heating and the DoE optimization under microwave conditions, as well as X‐ray diffraction data with refinement details, DFT data and tables on the statistical DoE data can be found in the Supporting Information.

Deposition Numbers “https://www.ccdc.cam.ac.uk/services/structures?id=https://doi.org/10.1002/chem.202502149” 2321746 (for **5**), 2321745 (for **8**) contain the supplementary crystallographic data for this paper. These data are provided free of charge by the joint Cambridge Crystallographic Data Centre and Fachinformationszentrum Karlsruhe “http://www.ccdc.cam.ac.uk/structures” Access Structures service.

DFT output files can be obtained free of charge from NOMAD (nomad‐lab.eu) with the upload ID iuDc63bVRriKhC79xdkOpQ.

## Supporting Information

The authors have cited additional references within the Supporting Information.^[^ ^14^, ^15^, ^16^, ^17^, ^18^, ^19^, ^20^, ^21^, ^22^, ^23^, ^24^, ^25^ ^]^

## Conflict of Interest

The authors declare no conflict of interest.

## Supporting information



Supporting Information

Supporting Information

## Data Availability

The data that support the findings of this study are available in the supplementary material of this article; the crystallographic data are available in the CCDC; and the DFT output files are available in NOMAD.

## References

[1] a) U. Wietelmann , J. Klett , Z. Anorg. Allg. Chem. 2018, 644, 194; 29540939 10.1002/zaac.201700394 PMC5838519

[2] a) A. A. Fyfe , A. R. Kennedy , J. Klett , R. E. Mulvey , Angew. Chem. Int. Ed. 2011, 50, 7776; 10.1002/anie.201103027 21739549

[3] a) V. H. Gessner , C. Däschlein , C. Strohmann , Chem. Eur. J. 2009, 15, 3320; 19260001 10.1002/chem.200900041

[4] For approaches that do not rely on the use of commercially available sovents, see

[5] a) V. H. Gessner , C. Strohmann , Angew. Chem. Int. Ed. 2007, 46, 8281; 10.1002/anie.200702116 17899584

[6] a) S. V. Kessar , P. Singh , Chem. Rev. 1997, 97, 721; 11848886 10.1021/cr950082n

[7] a) C. O. Kappe , A. Stadler , D. Dallinger , Microwaves in Organic and Medicinal Chemistry, Vol. 2., Wiley‐VCH, Weinheim 2012;

[8] C. O. Kappe , Angew. Chem. Int. Ed. 2004, 43, 6250. 10.1002/anie.200400655 15558676

[9] a) R. A. Finnegan , H. W. Kutta , J. Org. Chem. 1965, 30, 4138;

[10] a) T. Kottke , D. Stalke , Angew. Chem. Int. Ed. Engl. 1993, 32, 580;

[11] a) A. Münch , L. Knauer , H. Ott , C. P. Sindlinger , R. Herbst‐Irmer , C. Strohmann , D. Stalke , J. Am. Chem. Soc. 2020, 142, 15897;. 32811141 10.1021/jacs.0c06035

[12] Sartorius , MODDE 13.1, Sartorius Stedim Data Analytics AB, Göttingen (Germany), 2024.

[13] F. Becke , F. W. Heinemann , T. Rüffer , P. Wiegeleben , R. Boese , D. Bläser , D. Steinborn , J. Organomet. Chem. 1997, 548, 205.

[14] T. Kottke , D. Stalke , J. Appl. Cryst. 1993, 26, 615.

[15] Bruker, Apex3, Bruker AXS Inc., Madison (USA), 2018.

[16] Bruker, Apex4, Bruker AXS Inc., Madison (USA), 2021.

[17] O. V. Dolomanov , L. J. Bourhis , R. J. Gildea , J. A. K. Howard , H. Puschmann , J. Appl. Cryst. 2009, 42, 339. 10.1107/S0021889811041161 PMC3236671 22199401

[18] G. M. Sheldrick , Acta Cryst. 2015, A71, 3.

[19] G. M. Sheldrick , Acta Cryst. 2015, C71, 3.

[20] R. D. Dennington , T. A. Keith , J. M. Millam , GaussView 6.0., Semichem Inc., Shawnee Mission (USA), 2016.

[21] M. J. Frisch , G. W. Trucks , H. B. Schlegel , G. E. Scuseria , M. A. Robb , J. R. Cheeseman , G. Scalmani , V. Barone , G. A. Petersson , H. Nakatsuji , X. Li , M. Caricato , A. Marenich , J. Bloino , B. G. Janesko , R. Gomperts , B. Mennucci , H. P. Hratchian , J. V. Ortiz , A. F. Izmaylov , J. L. Sonnenberg , D. Williams‐Young , F. Ding , F. Lipparini , F. Egidi , J. Goings , B. Peng , A. Petrone , T. Henderson , D. Ranasinghe , V. G. Zakrzewski , J. Gao , N. Rega , G. Zheng , W. Liang , M. Hada , M. Ehara , K. Toyota , R. Fukuda , J. Hasegawa , M. Ishida , T. Nakajima , Y. Honda , O. Kitao , H. Nakai , T. Vreven , K. Throssell , J. A. Montgomery Jr. , J. E. Peralta , F. Ogliaro , M. Bearpark , J. J. Heyd , E. Brothers , K. N. Kudin , V. N. Staroverov , T. Keith , R. Kobayashi , J. Normand , K. Raghavachari , A. Rendell , J. C. Burant , S. S. Iyengar , J. Tomasi , M. Cossi , J. M. Millam , M. Klene , C. Adamo , R. Cammi , J. W. Ochterski , R. L. Martin , K. Morokuma , O. Farkas , J. B. Foresman , D. J. Fox , Gaussian 09, Revision E.01, Gaussian, Inc., Wallingford (USA), 2016.

[22] S. Grimme , WIREs Comput. Mol. Sci. 2011, 1, 211.

[23] P. Flükiger , H. P. Lüthi , S. Portmann , J. Weber , MOLEKEL 4.3, Swiss Center for Scientific Computing, Manno (Switzerland), 2000.

[24] J. Kleinheider , C. Schwab , C. Strohmann , Organometallics 2023, 42, 3173.

[25] Bruker, XP – Interactive molecular graphics. Version 5.1, Bruker AXS Inc., Madison, Wisconsin (USA), 1998.

