# Supplementary material for: Microwave Radiation for Organolithium Chemistry: Mechanistic Studies on the Direct α‐Metalation of a Tertiary Amine
Source: Chemistry. 2025 Aug 14;31(54):e02149. doi: 10.1002/chem.202502149 (PMC12462239; doi:10.1002/chem.202502149)
Supplement: Supplementary file 1 — Supporting Information [file CHEM-31-e02149-s001.pdf]

Supporting Information
©Wiley-VCH 2021
69451 Weinheim, Germany

Microwave Radiation for Lithium Chemistry: Mechanistic Studies on the Direct α-Metalation of a Tertiary Amine

Annika Schmidt,[§] Rebecca Scheel,[§] Andrea Ost, Lukas Brieger and Carsten Strohmann*

Abstract: Deprotonation reactions with lithium alkyls exhibit a high temperature dependency. However, the reactivity of lithium alkyls with conventionally used Lewis-bases limits their deprotonation capability of more challenging substrates like tertiary amines. Therefore, we have focused on the use of high but exactly set reaction temperatures by usage of microwave radiation in the deprotonation reaction of a tertiary amine in the absence of polar additives. Our study proves the robustness of lithium alkyls, like *t*-butyllithium and α-metalated tertiary amines under microwave conditions. A solid-state structure of the pre-lithiation aggregate and quantum chemical calculations offer an insight into the mechanism, revealing the substrate amine performs as Lewis-base for deaggregation itself. By our method, *N*-methylpiperidine was deprotonated without any intermediate or possibly toxic reaction steps, solvents or additives, solely with *t*-butyllithium in the microwave with 56% yield. By using *design of experiments* we identified the most important factors for a successful deprotonation, revealing that the closed reaction conditions under pressure and an excess of amine to be crucial. This investigation proves the high potential of microwave radiation in lithium chemistry, as it provides high but exact energy and introduces new possibilities for deprotonation reactions to overcome high kinetic reaction barriers.

Table of Contents

1 General Remarks **3**
2 Experimental Procedures **4**
2.1 Single crystal X-ray diffraction analysis 11
3 Results and Discussion **15**
3.1 Quantum chemical calculations 15
3.2 Design of Experiment 18

1 General Remarks

All reactions with oxygen- and moisture-sensitive compounds were performed under an atmosphere of argon in dried solvents, which were distilled prior to use. All other solvents and commercially available reagents, including the NMR solvents, were used without further purification.

The NMR spectra were measured on a *Bruker Advance 400* and on a *Bruker Advance 500* NMR spectrometer. All NMR spectra were recorded at room temperature (approx. 22 °C). Chemical shifts (δ in ppm) are referred to tetramethylsilane (TMS), with the deuterium signal of the solvent serving as internal lock and the residual solvent signal as additional reference [^{1}H-NMR: δ(C_6D_5H) = 7.16, δ($CHCl_3$) = 7.26; ^{13}CNMR: δ(C_6D_6) = 128.4, δ($CDCl_3$) = 77.0]. For the assignment of the multiplicities the following abbreviations were used: s = singlet, d = doublet, t = triplet, m = multiplet, br = broad signal.

GC/EI-MS analyses were obtained using an *Agilent 7890B GC system* (column: *Agilent HP-5MS*, 30 m, 0.25 mm, 0.25 µm) with an *Agilent 5977A Mass Selective Detector*.

The *vario MICRO cube* device from *elementar* was used for elemental analysis.

The *ReactIR 700* from *Mettler Toledo* was used. The probe, equipped with a *DiComp* (diamond-composite) sample head, is coupled to an AgX 6 mm x 1.5 m glass fiber (silver halide). The measurement was carried out in a wavenumber range from 3000 cm^{-1} to 650 cm^{-1} with a resolution of 4 wavenumbers. The scan option was set to autoselect in each case. The digital evaluation was carried out using the device's own *iC IR 7.1* software.

The *Discover SP* device from *CEM GmbH* was used for the microwave reactions. 10 mL reaction vessels made of borosilicate glass with silicone lids were used. The energy was regulated via a temperature controller. The pressure limit was 17.2 bar and the stirring power was set to “high”.

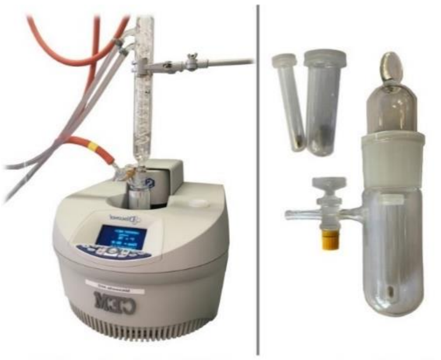

Figure S1: Overview of preparative techniques for inert microwave synthesis. Open reaction control with Schlenk flask in microwave and attached reflux condenser (left). Closed microwave vessels and/in uniquely designed Schlenk flask for preparation (right).

Suitable crystals of compounds **5** and **8** were covered with an inert oil (perfluoroalkylether) at –80 °C using the *X-TEMP 2*[1] device in combination with a *SMZ1279* stereomicroscope from *Nikon Metrology GmbH* and mounted on a *MicroMount* from *MiTeGen*. Crystal structure determination was accomplished on a *Bruker D8 Venture* four-circle diffractometer using a *PHOTON II CPAD* detector by *Bruker AXS GmbH*. X-ray radiation was generated by microfocus source IµS Mo (λ = 0.71073 Å) by *Incoatec GmbH* with HELIOS mirror optics and a single hole collimator by *Bruker AXS GmbH*. For the data collection, the programs APEX 3 Suite (v.2018.7-2)[2] and APEX4 Suite (v2021.10-0)[3] with the integrated programs SAINT (integration) and SADABS (adsorption correction) by *Bruker AXS GmbH* were used. The processing and finalization of the crystal structure was done with the program Olex2.[4] The crystal structure was solved with the ShelXT[5] structure solution program using Intrinsic Phasing and refined with the ShelXL refinement package using Least Squares minimization.[6] The non-hydrogen atoms were refined anisotropically. U_{eq} is defined as one third of the trace of the orthogonalized tensor U_{ij}. For the hydrogen atoms the standard values of the SHELXL program were used with U_{iso}(H) = –1.2 U_{eq}(C) for CH_2 and CH and with U_{iso}(H) = –1.5 U_{eq}(C) for CH_3.

The quantum chemical calculations were carried out using the following programs: The molecules, if they could not be transferred from molecular structures in the solid state, were modeled with the inferface *GaussView 6.0*[7] and a Gaussian Job File (gjf, input files) was created. The calculations were carried out with the programs *Gaussian 09 Revision E.01*.[8] All basic state structures were initially optimized without symmetry restrictions. A subsequent frequency calculation did not provide any imaginary frequencies for the minimum structures. The quantum chemical calculations were carried out using the basis sets 6-31+G(d,p) and the hybrid DFT functional *B3LYP*. An additional dispersion correction *D3* according to *Grimme* was used.[9] The visualization of the energy-optimized structures was carried out with the program *Molekel 4.32*.[10] The conditions chosen for the temperature were 298.15 K (room temperature) and for the pressure 1 bar (standard pressure).

2 Experimental Procedures

t-Butyllithium under microwave radiation

In our experiments, we observed the decomposition of *t*-butyllithium with small amounts of etheric solvents and for some charges of commercial *t*-butyllithium under microwave radiation. It is known, that the presence of alkoxides promotes the decomposition of butyl lithium,[11] which we assume to be reason for the partially observed decomposition.[12] In such cases of trace amounts of alkoxides being present, trapping of the decomposition products with trimethyltinchloride was possible.

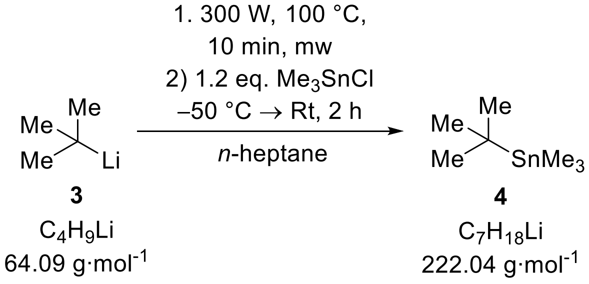

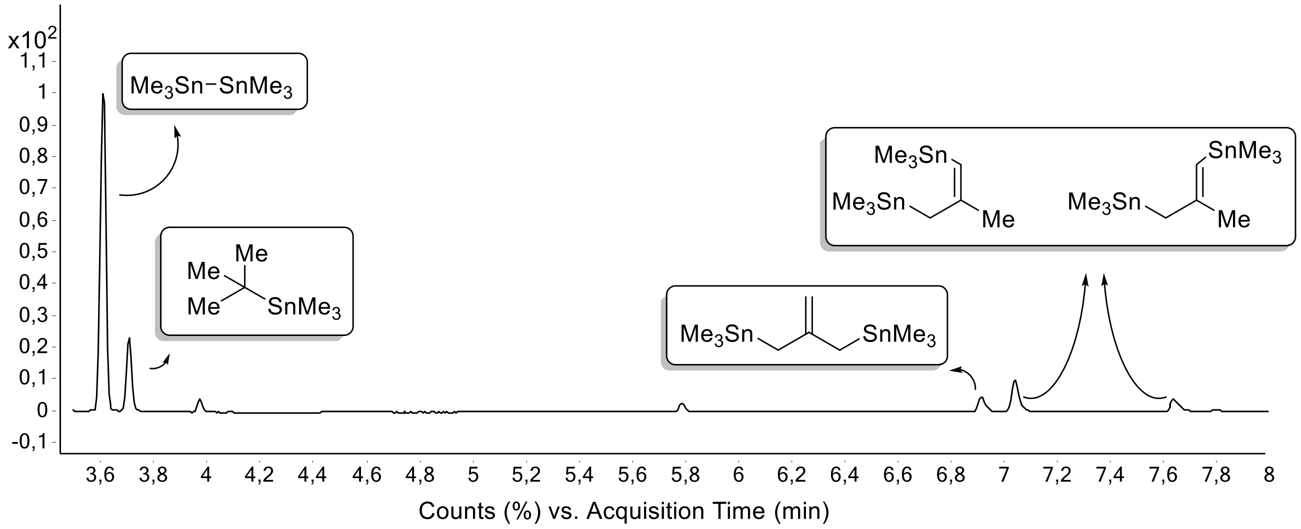

Figure S2: GC/MS chromatogram with decomposition products of *t*-butyllithium trapped with trimethyltinchloride from impure *t*-butyllithium.

However, in our research, we have also observed multiple reactions where decomposition of *t*-butyllithium has not been observed, especially for reaction mixtures when different aggregates like the reported dimeric structure **5** is formed. Additionally, also for pure *t*-butyllithium under the given conditions of this work, we observed no decomposition:

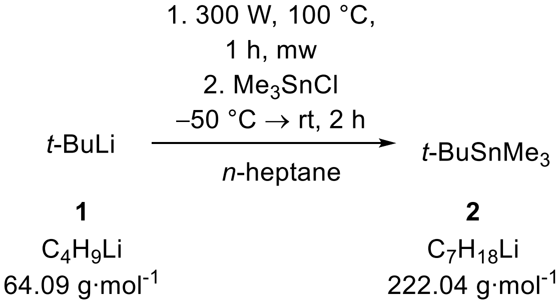

The lithium alkyl *t*-butyllithium (**3**, 1.00 eq., 2.00 mmol, 1.05 mL; 1.9 M in *n*-pentane) was added to an evacuated Schlenk flask and the contained *n*-pentane was removed via vacuum. Then *n*-heptane was added, the reaction solution was transferred to a microwave vial and heated by microwave radiation (100 °C, 300 W, 1 h). The reaction solution was then cooled to –50 °C and *tri*-methylchlorostannane (1.10 eq., 2.20 mmol, 437 mg) was added. After stirring for 2 h at room temperature, the reaction solution was analyzed by GC/EI-MS.

GC/EI-MS: [50 °C (0.5 min – 290 °C (2.0 min) with 20 °C/min], (70 eV, t_R = 3.47 min); *m/z* (%): 222 (10) $[(M)^+]$, 207 (20) $[(M–CH_3)^+]$, 164 (100) $[(SnMe_3)^+]$, 57 (60) $[(M–SnMe_3)^+]$.

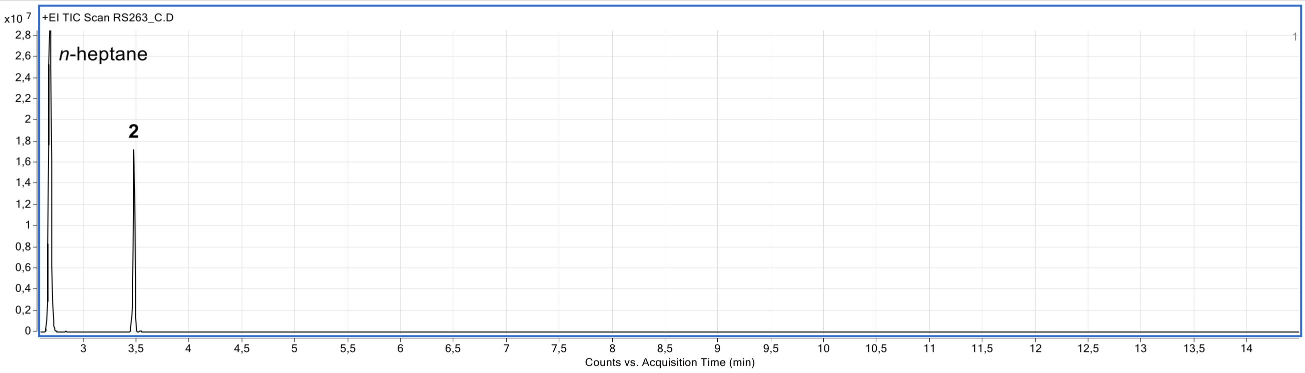

Figure S3: GC/EI-MS chromatogram of *tert*-butyllithium under microwave radiation after quenching with *tri*-methylchlorostannane.

Deprotonation under conventional heating

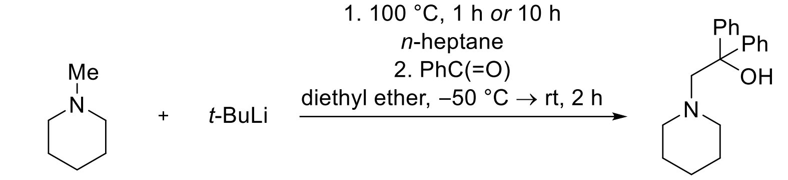

**1**	**3**	**7**
$C_6H_{13}N$	C_4H_9Li	$C_{19}H_{23}NO$
99.10 g·mol^{-1}	64.09 g·mol^{-1}	281.18 g·mol^{-1}

Tert-butyllithium (**3**, 1.00 eq., 4.00 mmol, 2.10 mL; 1.9 M in *n*-pentane) was added to an evacuated Schlenk flask and the contained *n*-pentane was removed via vacuum. Then *N*-methylpiperidine (**1**, 1.00 eq., 4.00 mmol, 0.49 mL) and *n*-heptane (2.00 mL) was added and the reaction solution was heated for 1 h or 10 h at 100 °C in an oil bath. The reaction solution was then cooled to –50 °C and benzophenone (1.20 eq., 4.80 mmol, 2.4 M in diethyl ether, 2 mL) was added. After stirring for 2 h at room temperature a NMR and a GC/MS sample was taken. NMR analysis revealed 5 % yield for the reaction heating for 1 h and 17 % yield for the reaction heating for 10 h.

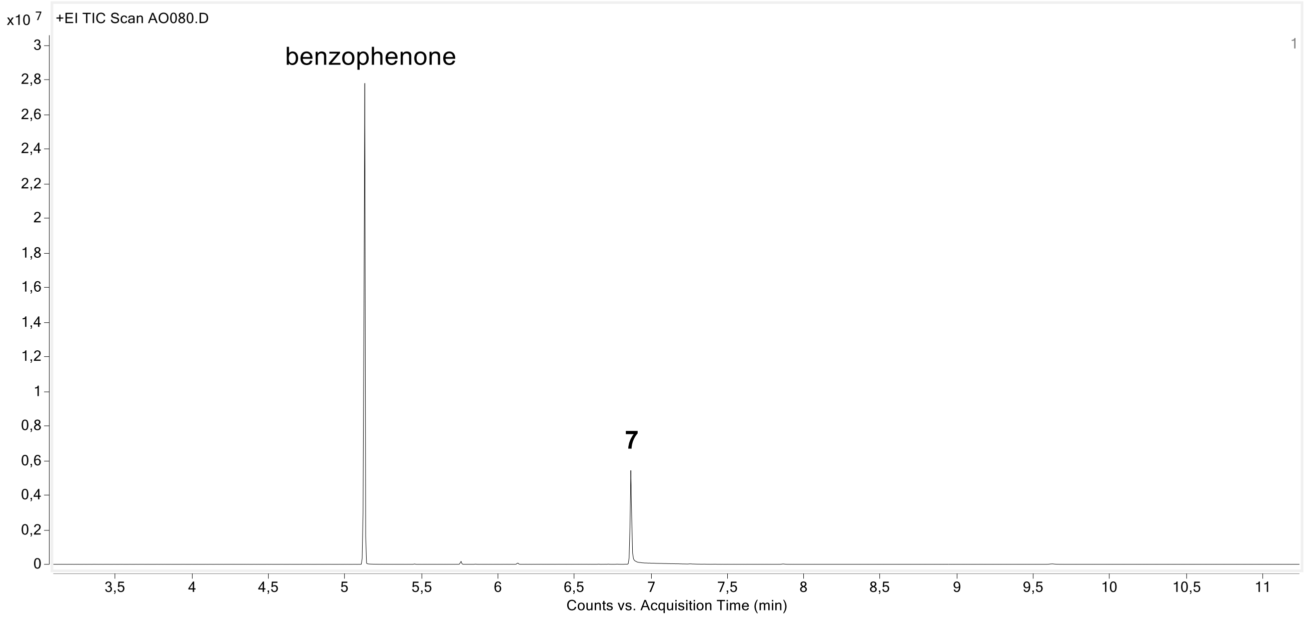

Figure S4: GC/EI-MS chromatogram of deprotonation of **1** under conventional heating for 1 hour after quenching with benzophenone.

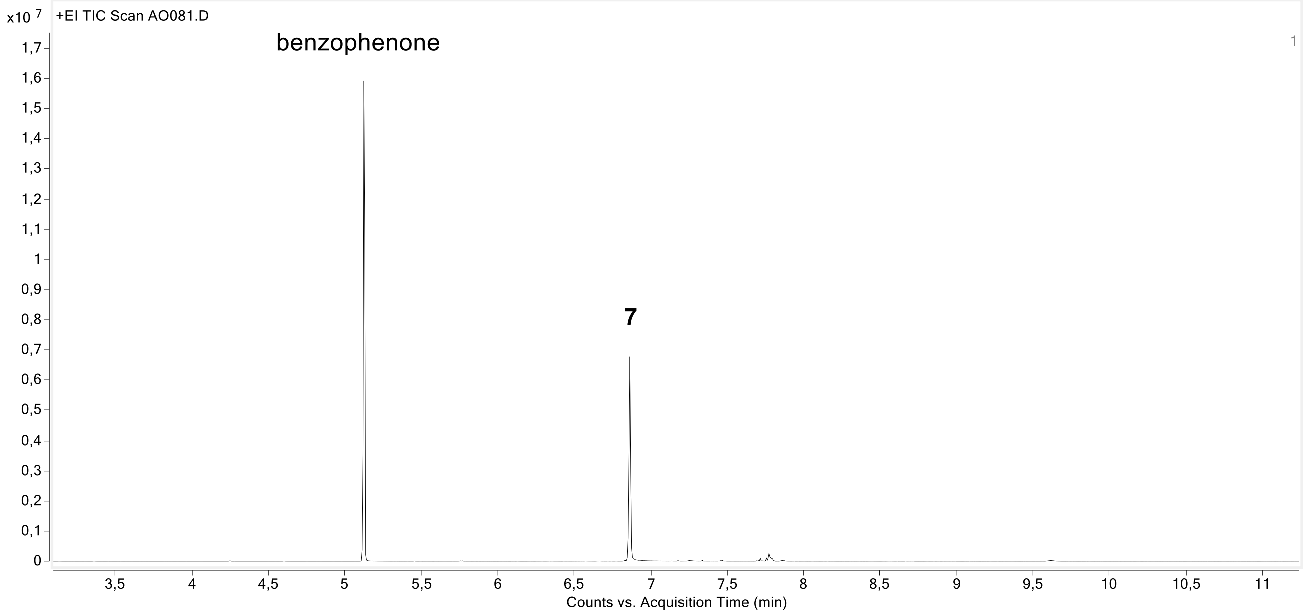

Figure S5: GC/EI-MS chromatogram of deprotonation of **1** under conventional heating for 10 h after quenching with benzophenone.

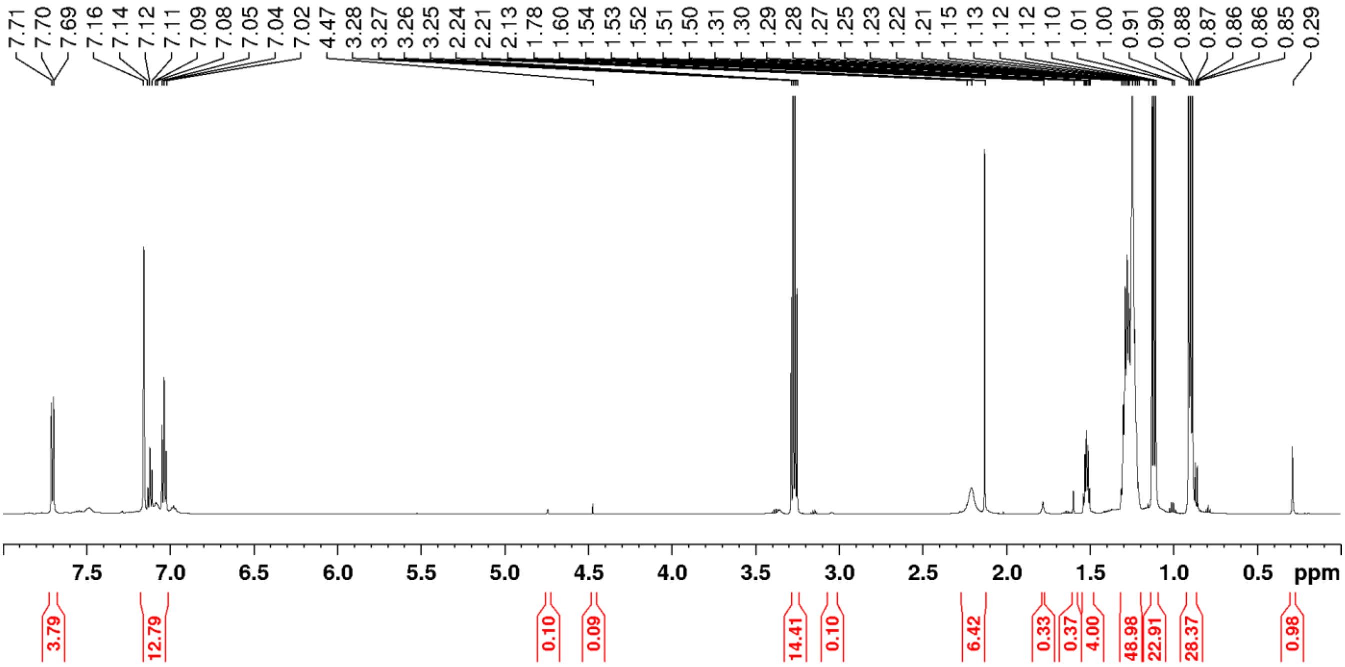

Figure S6: ^{1}H-NMR spectrum of deprotonation of **1** under conventional heating for 1 h after quenching with benzophenone.

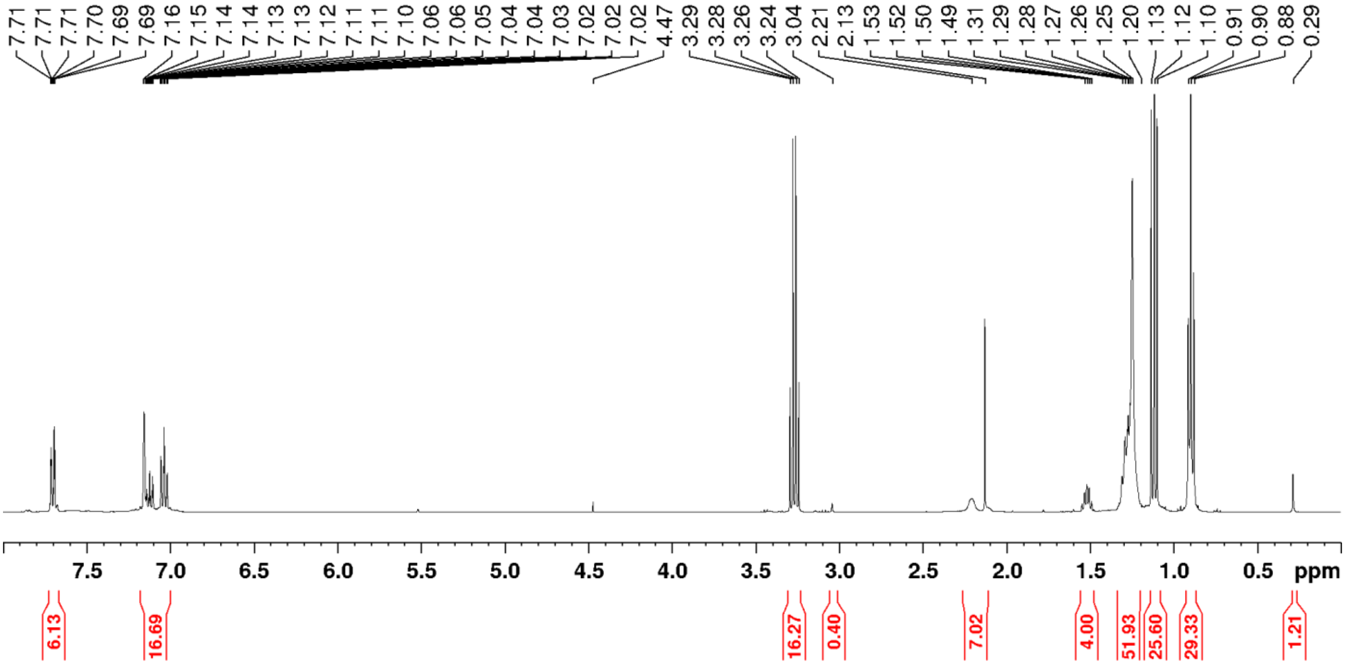

Figure S7: **^{1}H-**NMR spectrum of deprotonation of **1** under conventional heating for 10 h after quenching with benzophenone.

FT-IR-spectroscopic investigations of *N*-methylpiperidine

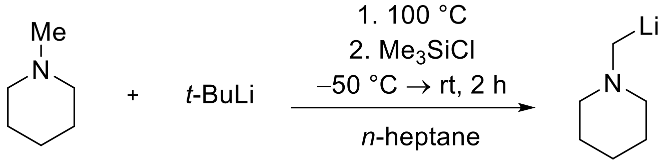

**1**	**3**	**2**
$C_6H_{13}N$	C_4H_9Li	$C_6H_{12}NLi$
99.10 g·mol^{-1}	64.09 g·mol^{-1}	105.11 g·mol^{-1}

N-heptane (2.00 mL) was added and the signals of *n*-heptane were identified *via* the IR probe and used as a reference. Subsequently, at room temperature, *t*-butyllithium (2.00 eq., 2.00 mmol, 1.05 mL, 1.9 M in *n*-pentane) and *N*-methylpiperidine (1.00 eq., 1.00 mmol, 0.10 mL) was added. The reaction solution was heated conventionally up to 100 °C and spectra were continuously recorded by the IR probe. The sensor was then removed, and the reaction solution was cooled to –50 °C. The reaction was treated with trimethylchlorosilane (2.10 eq., 2.10 mmol, 0.27 mL) and stirred for 2 h.

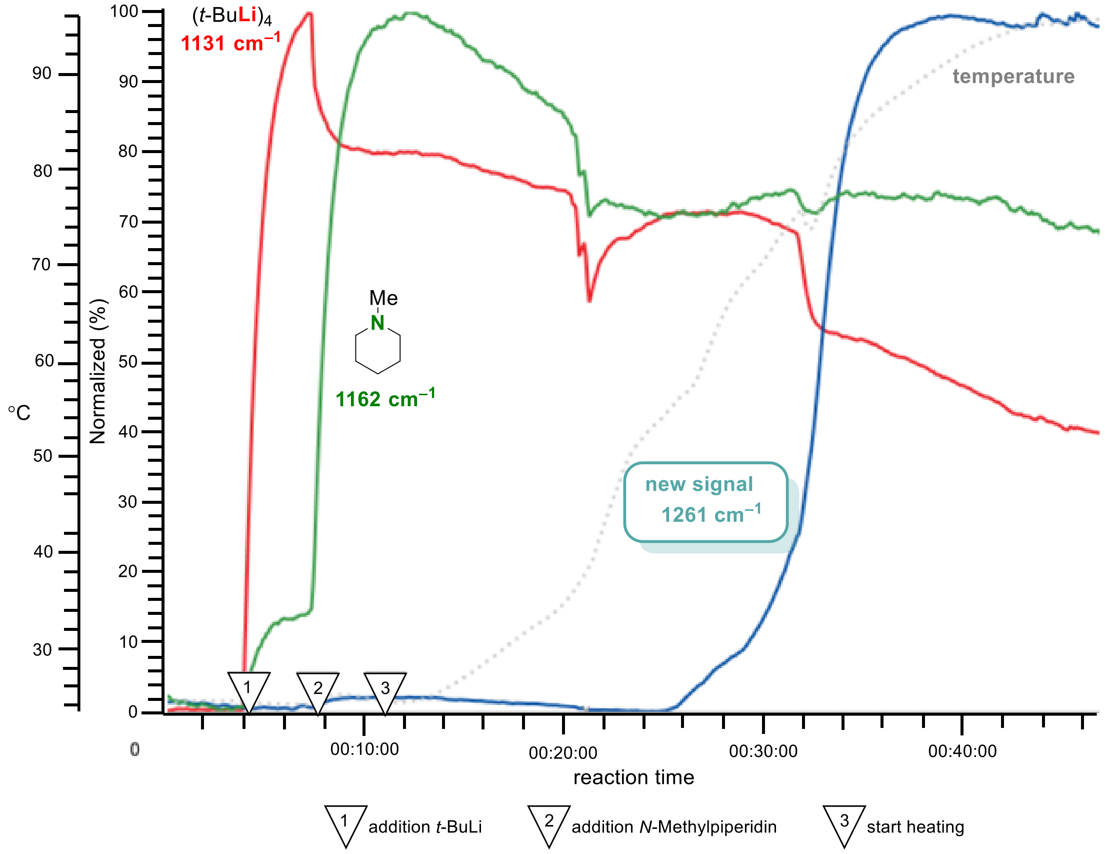

Figure S8: Course of the relevant IR-bands of the FT-IR-spectroscopic investigation of the deprotonation of *N*-methylpiperidine (**1**) with *t*-BuLi.

Table S1: Data description of figure S8.

Trend	Color	Units
t-BuLi peak at 1131 cm^{-1}		Height
N- methylpiperidine peak at 1162 cm^{-1}		Height
Product peak at 1261 cm^{-1}		Height
Probe Temp		Deg C

Deprotonation reaction of amines using microwave radiation

General procedures:

1) under Reflux

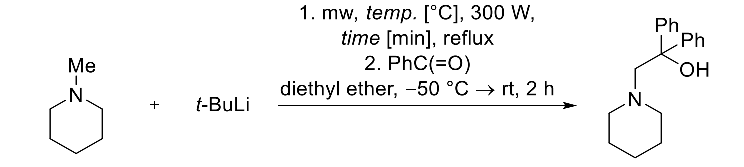

**1**	**3**	**7**
$C_6H_{13}N$	C_4H_9Li	$C_{19}H_{23}NO$
99.10 g·mol^{-1}	64.09 g·mol^{-1}	281.18 g·mol^{-1}

Tert-butyllithium (**3**, 1.00 eq., 4.00 mmol, 2.10 mL; 1.9 M in *n*-pentane) was added to an evacuated Schlenk flask and the contained *n*-pentane was removed via vacuum. Then *N*-methylpiperidine (**1**, 1.00–5.00 eq., 4.00–20.00 mmol, 0.49–2.45 mL) was added and the reaction solution was heated by microwave radiation (90–120 °C, 300 W, 5–120 min, under reflux). The reaction solution was then cooled to –50 °C and benzophenone (1.20 eq., 4.80 mmol, 2.4 M in diethyl ether, 2.00 mL) was added. After stirring for 2 h at room temperature a NMR and GC/MS sample was taken.

2) closed microwave vessel

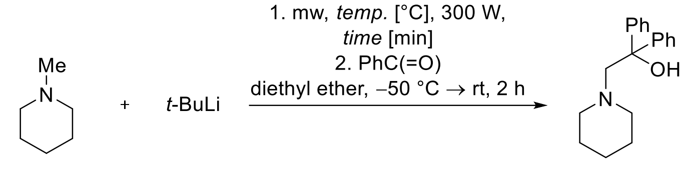

1 $C_6H_{13}N$ 99.10 g·mol^{-1} **3** C_4H_9Li 64.09 g·mol^{-1} **7** $C_{19}H_{23}NO$ 281.18 g·mol^{-1}

Tert-butyllithium (**3**, 1.00 eq., 4.00 mmol, 2.10 mL; 1.9 M in *n*-pentane) was added to an evacuated Schlenk flask and the contained *n*-pentane was removed via vacuum. Then *N*-methylpiperidine (**1**, 1.00–5.00 eq., 4.00–20.00 mmol, 0.49–2.45 mL) was added and the reaction solution was transferred to a microwave vial. The microwave vial was closed and the reaction solution was heated by microwave radiation (90–120 °C, 300 W, 5–120 min). The reaction solution was then cooled to –50 °C and benzophenone (1.20 eq., 4.80 mmol, 2.4 M in diethyl ether, 2.00 mL) was added. After stirring for 2 h at room temperature a NMR and a GC/MS sample was taken.

Analytics of 7

GC/EI-MS [80 °C (1 min) – 270 °C (5.5 min) with 40 °C/min], (70 eV, t_R = 6.869 min); *m/z* (%): 98 (100) [$(NC_6H_{12})^+$], 77 (19) [$(Ph)^+$].

^{1}H-NMR (400.25 MHz, C_6D_6, 25 °C, TMS, ppm): δ = 7.69 [m, 4H; aromatic *H*], 7.17 [m, 2H; aromatic *H*], 7.02 [m, 4H; aromatic *H*], 5.45 [br.s., 1H; O*H*], 3.04 [s, 2H; CCH_2N], 2.10 [m, 4H; $NCH_2CH_2CH_2$], 1.16 [m, 4H; $NCH_2CH_2CH_2$], 1.06 [m, 2H; $NCH_2CH_2CH_2$].

{^{1}H}^{13}C-NMR (100.6 MHz, C_6D_6, 25 °C, TMS, ppm): δ = 148.9 [s, 1C, C_{ipso}], 128.7 [s, 2C; C_{meta}], 127.0 [s, 1C; C_{para}], 126.4 [s, 2C; C_{ortho}], 75.5 [s, 1C; *C*OH], 69.1 [s, 1C; N*C*H$_2$COH], 56.3 [s, 2C; N*C*H$_2$CH$_2$CH$_2$], 26.7 [s, 2C; NCH$_2$*C*H$_2$CH$_2$], 24.4 [s, 1C; NCH$_2$CH$_2$*C*H$_2$].

Elemental analysis calculated: %C 81.10 %H 8.24 %N 4.98
measured: %C 81.5 %H 8.0 %N 4.7

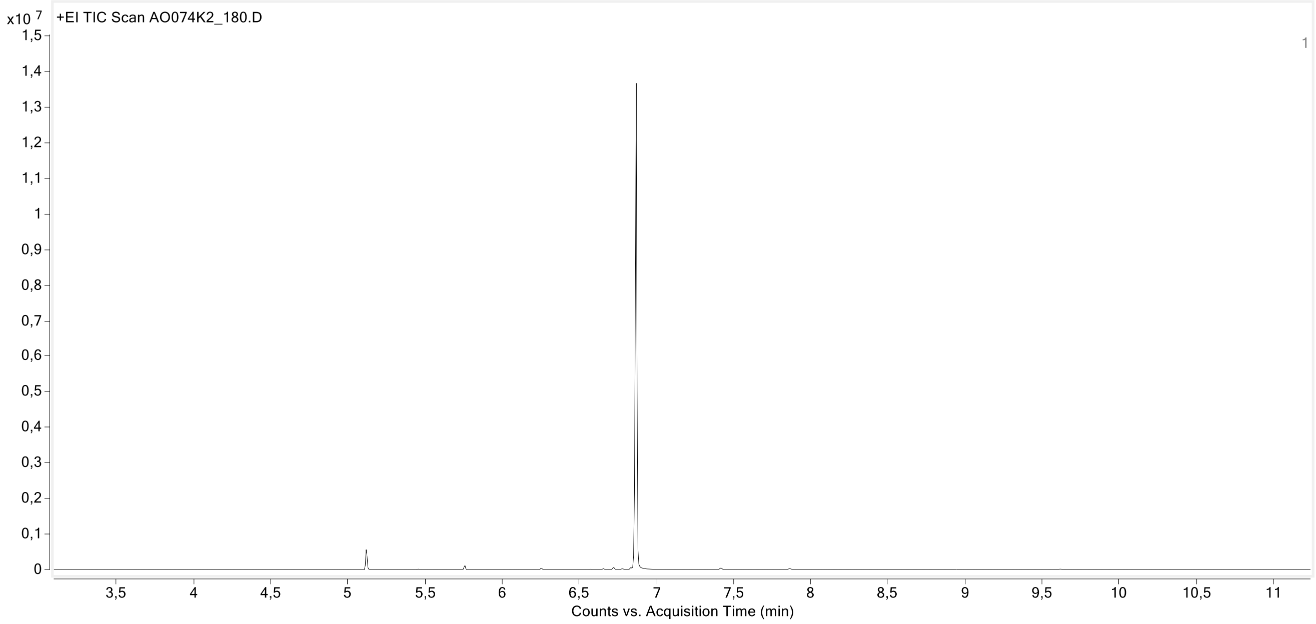

Figure S9: GC/MS-chromatogram of **7**.

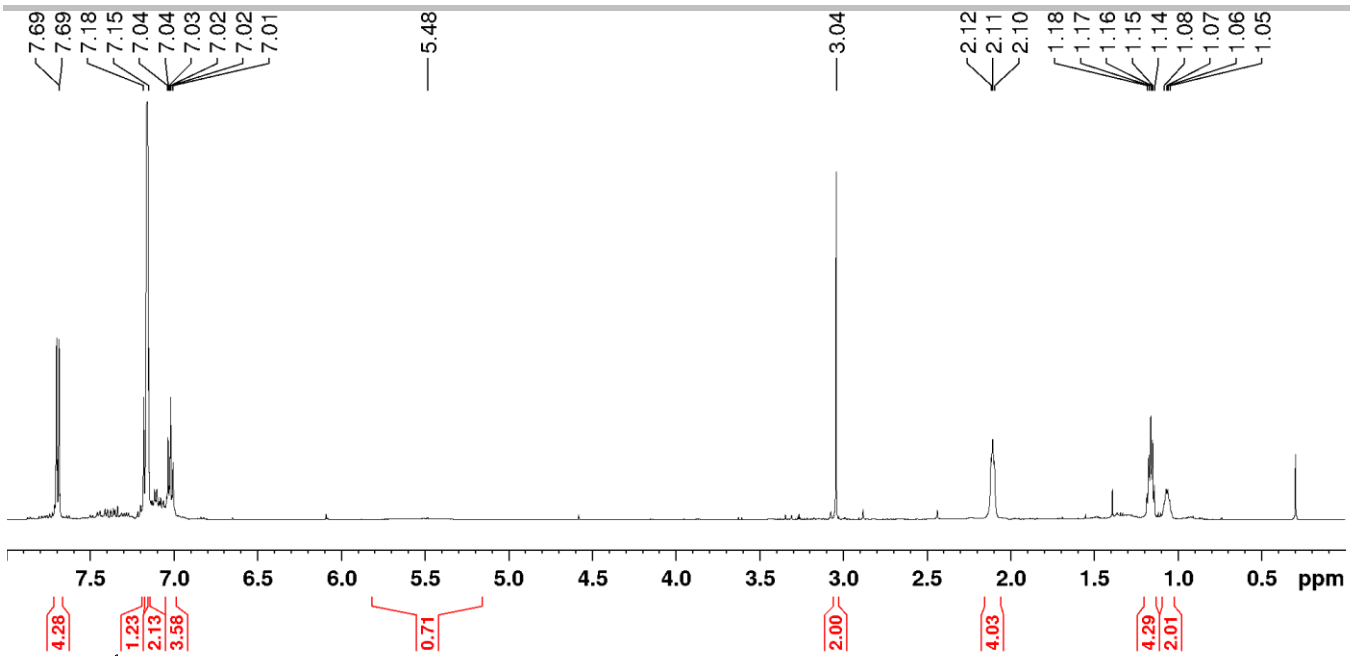

Figure S10: ^{1}H-NMR spectrum of **7**.

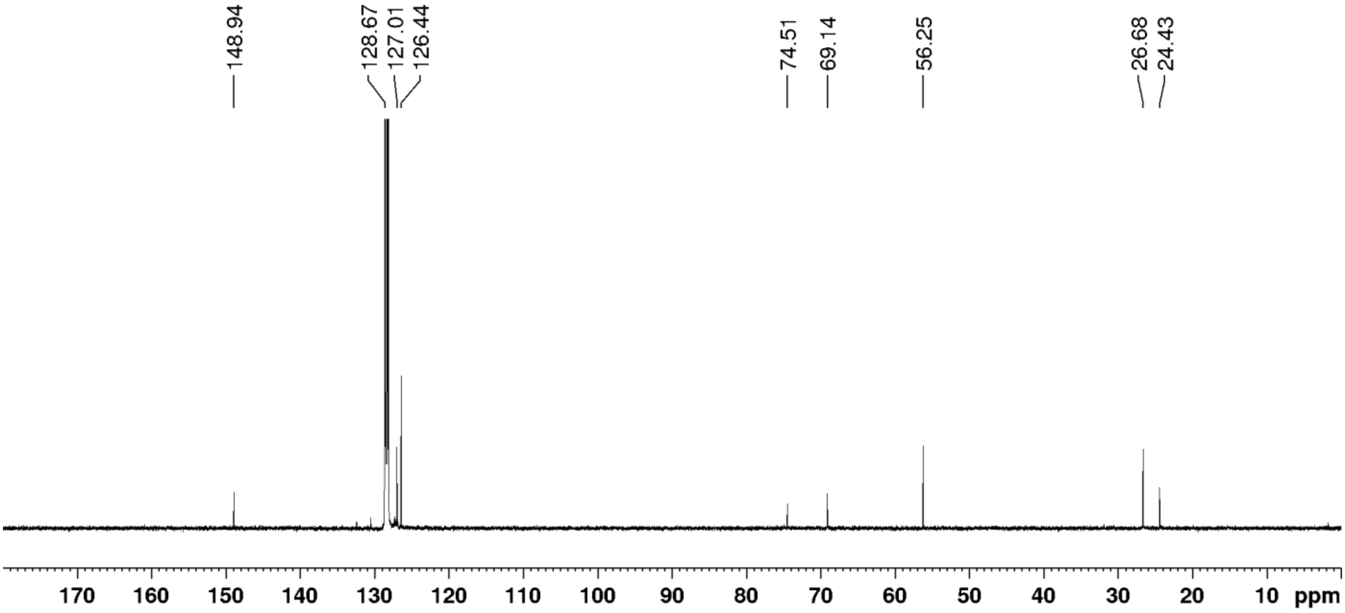

Figure S11: ^{13}C-NMR spectrum of **7**.

2.1 Single crystal X-ray diffraction analysis

Crystallographic data for the structures of **5** and **8** have been deposited with the Cambridge Crystallographic Data Centre as supplementary publication numbers 2321746 (for **5**) and 2321745 (for **8**) respectively. Copy of these data can be obtained, free of charge, on application to CCDC, 12 Union Road, Cambridge CB2 IEZ, UK, fax: 144-(0)1223-336033 or email: deposit@ccdc.cam.ac.uk.

Crystallization procedures
Crystallization of compound 5

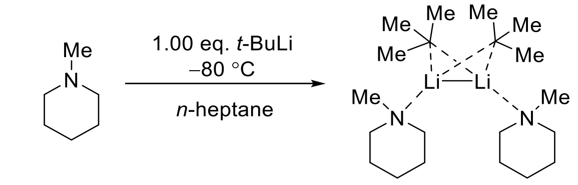

1 $C_6H_{13}N$ 99.10 g·mol^{-1} **5** $C_{20}H_{44}Li_2N_2$ 326.38 g·mol^{-1}

N-methylpiperidine (**1**, 1.00 eq., 2.00 mmol, 0.24 mL) was placed in *n*-heptane (2.00 mL). Subsequently, at –80 °C, *tert*-butyllithium (1.00 eq., 2.00 mmol, 1.05 mL; 1.9 M in *n*-pentane) was added. The product **5** crystallizes within 2 days at –80 °C.

^{1}H-NMR (600 MHz; C_6D_6, 25 °C, TMS, ppm): δ = 2.20 [s, 4H; NC*H*$_2$CH$_2$CH$_2$], 2.10 [s, 3H; NC*H*$_3$], 1.48 – 1.52 [m, 4H; NCH$_2$C*H*$_2$CH$_2$], 1.22 [m, 2H; NCH$_2$CH$_2$C*H*$_2$], 0.97 [s, 9H; C(C*H*$_3$)$_3$].
{^{1}H}^{13}C-NMR (150.94 MHz, C_6D_6, 25 °C, TMS, ppm): δ = 57.4 [2C; N*C*H$_2$CH$_2$CH$_2$], 47.6 [1C; N*C*H$_3$], 30.0 [3C; C(*C*H$_3$)$_3$], 26.9 [2C; NCH$_2$*C*H$_2$CH$_2$], 23.2 [1C; NCH$_2$CH$_2$*C*H$_2$], 14.6 [1C; *C*(CH$_3$)$_3$].
^{7}Li-NMR (233.3 MHz, C_6D_6, 25 °C, LiCl, ppm): δ = 1.5 [1Li].

Regarding **5** DOSY-experiments were carried out. Measurement at room temperature in deuterated benzene reveals an exchange process due to very broad signals and no clear identification. To get a closer insight into the exchanging species measurements at lower temperatures were carried out, but the reaction solution at –30 °C shows a precipitate and therefore no clear identification is possible. Therefore, we assume that in the reaction solution might be an exchange between the dimeric form and the pure tetrameric butyllithium compound and the amine.

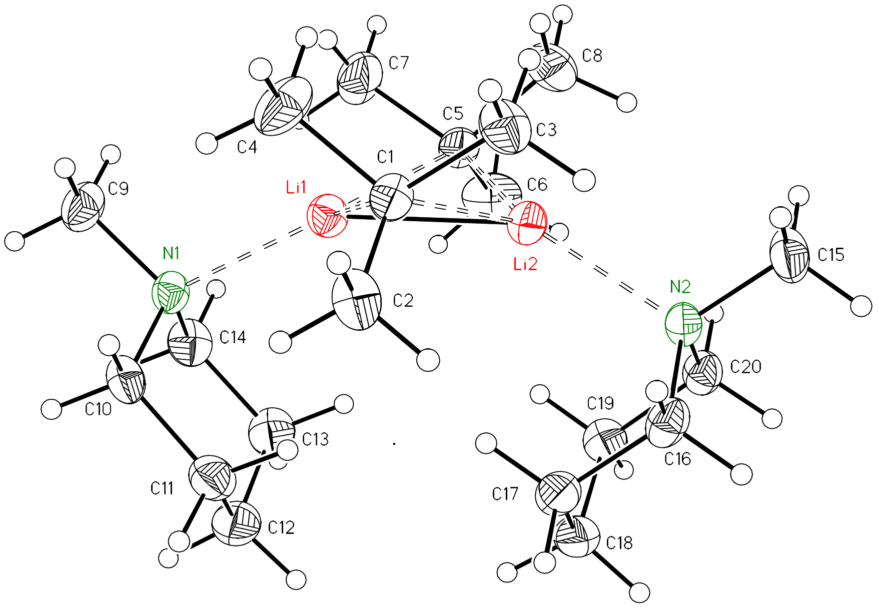

Figure S12: ORTEP[13] plot and numbering scheme of compound **5**. Displacement ellipsoids are drawn at 50% probability level. Selected bond lengths [A] and angles [°]: Li1–N1 2.137(2), N1–C1 1.467(16), N2–C7 1.474(16), N2–Li2 2.160(2), Li1–Li2 2.347(3), Li1–C13 2.243(3) Li2–C13 2.233(2) Li1–C17 2.212(2) Li2–C17 2.244(3), Li1–N1–C1 111.11(10), Li1–C17–Li2 63.55(9), Li1–Li2–N2 152.30(12).

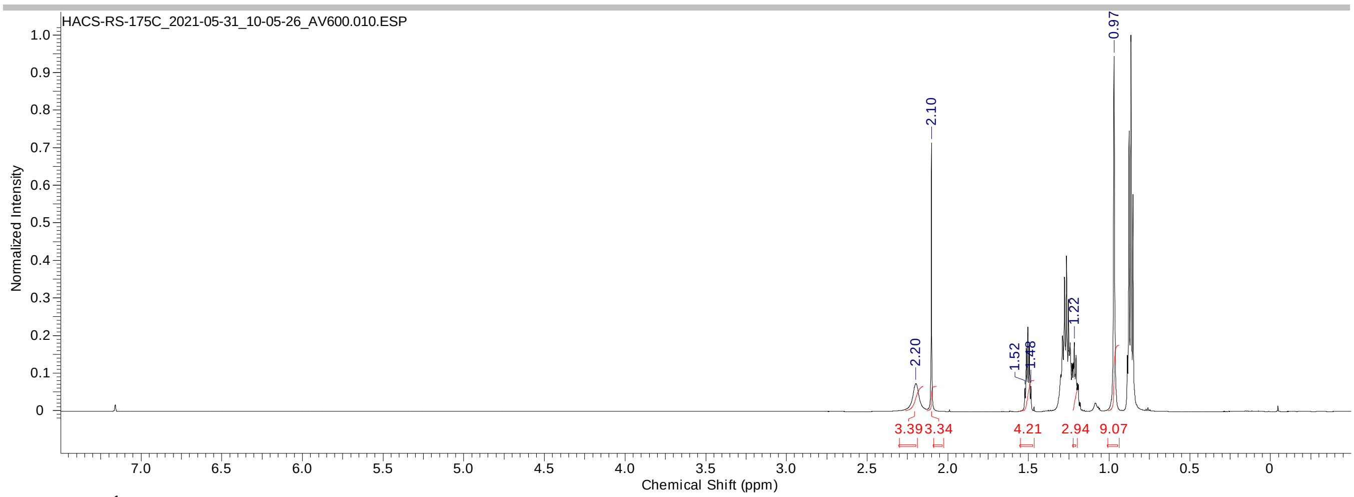

Figure S13: ^{1}H-NMR spectrum of **5**.

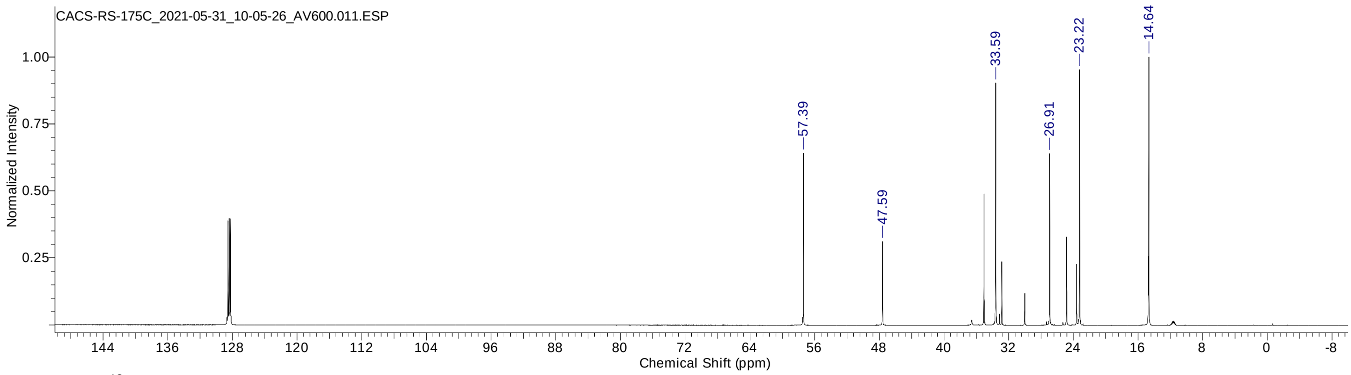

Figure S14: ^{13}C-NMR spectrum of **5**; remaining peaks can be assigned to *n*-heptane.

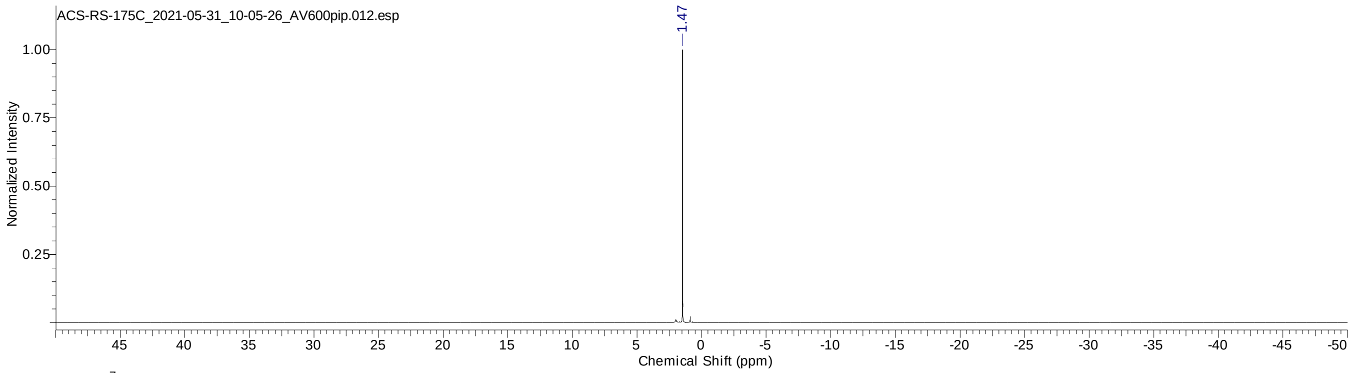

Figure S15: ^{7}Li-NMR spectrum of **5**.

Crystallization of compound 8[14]

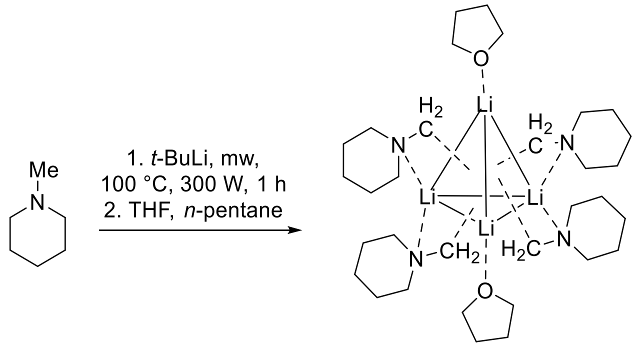

**1**	**8**
$C_6H_{13}N$	$C_{36}H_{76}Li_4N_4O_2$
99.10 g·mol^{-1}	624.66 g·mol^{-1}

T-butyllithium (**3**, 1.00 eq., 4.00 mmol, 2.10 mL; 1.9 M in *n*-pentane) was added and the contained *n*-pentane was removed via vacuum. Then *N*-methylpiperidine (**1**, 2.00 mL) was added and the reaction solution was transferred to a microwave vial and heated by microwave radiation (100 °C, 300 W, 1 h, under reflux). The reaction solution was dried under vacuum and at –78 °C tetrahydrofuran (0.50 mL) and *n*-pentane (1.00 mL) were added and stored at –80 °C for 7 days.

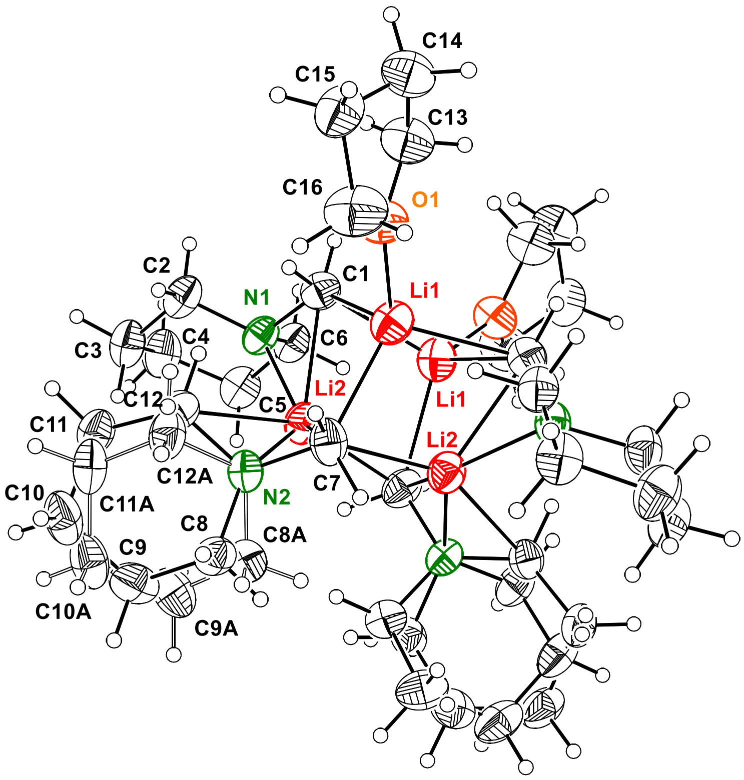

Figure S16: ORTEP[13] plot and numbering scheme of compound **8**. Displacement ellipsoids are drawn at 50% probability level. Selected bond lengths [A] and angles [°]: Li1–Li2 2.626(7), Li1–Li1^{1} 2.412(9), Li1–O1 1.948(5), Li1–C1 2.286(5), C1–N1 1.512(4), C7–N2 1.507(3), N2–Li2 2.058(5), N1–Li2 2.238(6), C7–Li1 2.262(6), Li1–C1–Li2 70.94(19), N1–C1–Li1 133.3(2), C1–N1–Li2 76.0(2).

Table S2: Crystal data and structure refinement for **5** and **8**.

**Compound**	**5**	**8**
Empirical formula	$C_{20}H_{44}Li_2N_2$	$C_{16}H_{32}Li_2N_2O$
Formula weight [g·mol^{-1}]	326.45	282.31
Temperature/K	100.0	100.00
Crystal System	orthorhombic	orthorhombic
Space Group	$P2_12_12_1$	*P*bcn
a [Å]	9.1567(3)	17.001(5)
b [Å]	15.4688(5)	10.939(3)
c [Å]	15.8126(5)	19.516(6)
α [°]	90	90
β [°]	90	90
γ [°]	90	90
Volume [Å^3]	2239.75(13)	3629.4(19)
Z	4	8
Density (calculated) ρ_{calc} [g·cm^{-3}]	0.968	1.033
Absorption coefficient μ [mm^{-1}]	0.390	0.467
F(000)	736.0	1248.0
Crystal Size [mm^3]	0.900 × 0.388 × 0.242	0.252 × 0.2 × 0.174
Radiation	CuK*α* (*λ* = 1.54178)	CuK*α* (*λ* = 1.54178)
Index ranges	–11 ≤ h ≤ 11<br>–19 ≤ k ≤ 19<br>–19 ≤ l ≤ 14	-20 ≤ h ≤ 19,<br>-13 ≤ k ≤ 13,<br>-20 ≤ l ≤ 23
Reflections collected	33358	39350
Independent reflections	4724 [R_{int} = 0.0359,<br>R_{sigma} = 0.0166]	3271 [R_{int} = 0.0511,<br>R_{sigma} = 0.0194]
Data / restraints / parameter	4724/0/269	3271/11/252
Goodness-of-fit an F^2	1.070	1.145
Final *R* indices [I≥2σ (I)]	R_1 = 0.0300,<br>wR_2 = 0.0854	R_1 = 0.0733, wR_2 =<br>0.1645
R indices [all data]	R_1 = 0.0303,<br>wR_2 = 0.0856	R_1 = 0.0784, wR_2 =<br>0.1669
Largest diff. Peak and hole [e Å^{-3}]	0.16/–0.13	0.19/-0.21
Absolute structure parameter	–0.06(9)	-

3 Results and Discussion

3.1 Quantum Chemical Calculations

In the DFT calculations, explicit "solvent" effects were taken into account by including the amine ligands, as the first coordination sphere of the lithium alkyls was modelled based on the solid-state dimer structure **5**. Since the reaction was carried out either without solvent or in the apolar solvent *n*-heptane, the omission of implicit solvation models is adequate, as no significant electrostatic contributions from the solvent are expected.

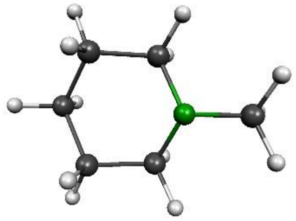

Figure S17: Calculated structure of *N*-methylpiperidine (**1**) [B3LYP/6-31+g(d,p);gd3].

Table S3: Standard orientation of *N*-methylpiperidine (**1**) [B3LYP/6-31+g(d,p);gd3], E_{SCF}= –291.257520782 H, ΔH = –291.062997 H; dipole moment: 0.4792 D.

atomic symbol	x	y	z
N	0.973027	0.000028	-0.311621
C	2.397831	0.000013	-0.012176
H	2.608968	-0.000189	1.076508
H	2.869597	0.886296	-0.449137
H	2.869604	-0.886126	-0.449429
C	0.316432	1.210855	0.186876
H	0.850399	2.080212	-0.213757
H	0.386605	1.268873	1.295253
C	-1.157122	1.257363	-0.229294
H	-1.211647	1.324634	-1.323383
H	-1.622768	2.162187	0.179824
C	-1.897748	0.000046	0.246483
H	-1.943307	0.00017	1.345302
H	-2.933098	-0.00003	-0.114563
C	-1.157175	-1.257355	-0.229119
H	-1.622733	-2.162076	0.180367
H	-1.212061	-1.324878	-1.323163
C	0.316458	-1.210903	0.186695
H	0.386893	-1.269187	1.295036
H	0.850298	-2.080184	-0.214293

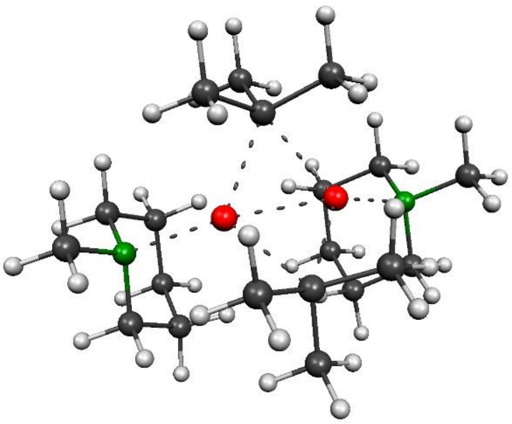

Figure S18: Calculated structure of *N*-methylpiperidine-*t*-BuLi dimer **5** [B3LYP/6-31+g(d,p);gd3].

Table S4: Standard orientation of *N*-methylpiperidine-*t*-BuLi dimer **5** [B3LYP/6-31+g(d,p);gd3], E_{SCF}= –913.372879638 H, ΔH = –912.721418 H; dipole moment: 4.3369 D.

atomic symbol	x	y	z
N	-3.03396500	0.08000800	0.00007400
N	3.03426700	0.07959700	0.00038200
C	-0.00065500	-1.51962100	-1.82061300
C	1.22886700	-2.44404900	-1.93636900
H	1.29494700	-3.17680900	-1.11831400
H	2.17743200	-1.88755500	-1.95666700
H	1.21893000	-3.03932400	-2.87436100

C	0.00236600	-0.61351500	-3.06035600
H	0.00134900	-1.19858300	-4.00631300
H	0.88493400	0.03790000	-3.10417900
H	-0.87679600	0.04249400	-3.10489800
C	-1.23322200	-2.43951700	-1.93959000
H	-2.17937800	-1.87929400	-1.96434000
H	-1.30543800	-3.17089500	-1.12070900
H	-1.22194200	-3.03636000	-2.87656700
C	0.00011500	-1.52281200	1.81874200
C	-1.23114900	-2.44478800	1.93549800
H	-1.22013400	-3.04223800	2.87209900
H	-1.30096000	-3.17564100	1.11597900
H	-2.17834600	-1.88620300	1.95945400
C	0.00068100	-0.61785100	3.05931300
H	-0.88017400	0.03580700	3.10451300
H	0.88155600	0.03584800	3.10369800
H	0.00111700	-1.20378400	4.00474400
C	1.23086800	-2.44549000	1.93496000
H	2.17859200	-1.88771900	1.95693800
H	1.29907300	-3.17736800	1.11627500
H	1.22062000	-3.04176500	2.87232800
C	4.13302100	-0.90230300	0.00052400
H	5.11896900	-0.40722600	-0.00003000
H	4.06637500	-1.53901300	-0.88525500
H	4.06698800	-1.53809700	0.88698600
C	3.12909500	0.92553400	-1.21473600
H	3.06059600	0.27210400	-2.09001200
H	4.12029600	1.41628100	-1.24650700
C	2.02933400	1.98773200	-1.25281900
H	1.05796600	1.48515700	-1.31959900
H	2.14157700	2.58599700	-2.16417600
C	2.07292300	2.86979100	-0.00004500
H	2.99747600	3.46445400	-0.00038600
H	1.23798300	3.57967700	-0.00009700
C	2.02962600	1.98845200	1.25326700
H	2.14281300	2.58724000	2.16417400
H	1.05778600	1.48692700	1.32088800
C	3.12888100	0.92568700	1.21542300
H	4.12024700	1.41608700	1.24745900
H	3.05984300	0.27240500	2.09076100
C	-4.13242900	-0.90221300	-0.00141200
H	-4.06596200	-1.53972100	0.88379800
H	-4.06556600	-1.53707800	-0.88846200
H	-5.11854100	-0.40746900	-0.00088200
C	-3.12824700	0.92708700	-1.21431800
H	-4.11960100	1.41752100	-1.24625300
H	-3.05899200	0.27441000	-2.09009700
C	-2.02896100	1.98983400	-1.25096200
H	-2.14175500	2.58938200	-2.16141600
H	-1.05712700	1.48829500	-1.31854300
C	-2.07265900	2.87015100	0.00307500
H	-1.23771800	3.58003700	0.00400400
H	-2.99721300	3.46481300	0.00361500
C	-2.02948000	1.98706400	1.25512400
H	-2.14193600	2.58458700	2.16694100
H	-1.05820300	1.48429400	1.32174400
C	-3.12924000	0.92491300	1.21585600
H	-3.06090600	0.27080100	2.09063300
H	-4.12049000	1.41555600	1.24776700
Li	-1.14403000	-0.98322300	-0.00051300
Li	1.14376100	-0.98266900	-0.00058500

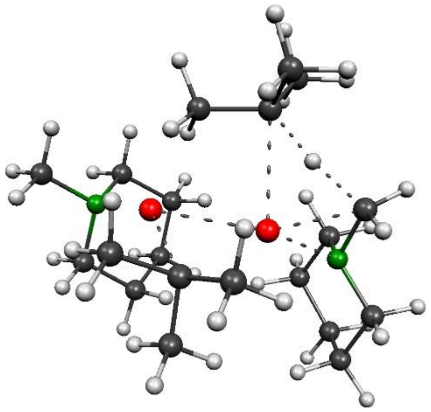

Figure S19: Calculated structure of transition state of deprotonation of *N*-methylpiperidine by *t*-BuLi in dimeric aggregate [B3LYP/6-31+g(d,p);gd3].

Table S5: Standard orientation of transition state of deprotonation of *N*-methylpiperidine by *t*-BuLi in dimeric aggregate [B3LYP/6-31+g(d,p);gd3], E_{SCF}=–913.313465206 H, ΔH = –912.668247 H; one imaginary frequency.

atomic symbol	x	y	z
N	2.644392	-0.241005	-0.085014
N	-3.124015	-0.167165	-0.159978
C	0.695782	2.63206	-1.255554
C	-0.808609	2.504319	-1.000836
H	-1.068348	2.706903	0.051367
H	-1.187931	1.517041	-1.309527
H	-1.40663	3.234073	-1.586531
C	0.954626	2.444305	-2.751014
H	0.47757	3.230968	-3.365249
H	0.572353	1.478879	-3.113953
H	2.030293	2.46671	-2.969042
C	1.16214	4.019163	-0.811161
H	2.245899	4.128325	-0.95043
H	0.956278	4.192329	0.254922
H	0.673884	4.835536	-1.375119
C	-0.219275	0.825856	2.265912
C	1.025912	1.598603	2.747052
H	1.030507	1.735839	3.845495
H	1.10156	2.60337	2.306024
H	1.968977	1.071301	2.526091
C	-0.235483	-0.530112	2.990947
H	0.610804	-1.163952	2.697421
H	-1.148744	-1.107117	2.788113
H	-0.18377	-0.414992	4.09372
C	-1.447371	1.628888	2.745185
H	-2.410614	1.159966	2.469442
H	-1.462534	2.658012	2.35741
H	-1.490027	1.713305	3.84879
C	-4.250732	0.743849	0.117596
H	-5.211606	0.312993	-0.207266
H	-4.099431	1.690255	-0.410762
H	-4.312701	0.949301	1.189907
C	-3.068072	-0.450739	-1.618155
H	-2.9689	0.502443	-2.146893
H	-4.024443	-0.903336	-1.937506
C	-1.915702	-1.393256	-1.969388
H	-0.957968	-0.907644	-1.739031
H	-1.921883	-1.574921	-3.049844
C	-2.032912	-2.707548	-1.187525
H	-2.926755	-3.251682	-1.523494
H	-1.171712	-3.352398	-1.388296
C	-2.147007	-2.412467	0.313033
H	-2.315514	-3.331742	0.884816
H	-1.20575	-1.982416	0.677439
C	-3.29088	-1.434982	0.596819
H	-4.256426	-1.900725	0.327872
H	-3.332953	-1.189951	1.663714
C	3.052506	1.171712	-0.330763
H	3.519248	1.55767	0.587552
H	1.782758	1.867759	-0.735711
H	3.806009	1.234337	-1.134793
C	2.264674	-0.885607	-1.351191
H	3.148919	-0.955807	-2.016457
H	1.5435	-0.240826	-1.863305
C	1.684286	-2.283948	-1.122583
H	1.417009	-2.733293	-2.087402
H	0.760829	-2.191315	-0.537846
C	2.674931	-3.171164	-0.357872
H	2.22932	-4.14817	-0.13409
H	3.554575	-3.358194	-0.990001
C	3.117493	-2.465237	0.931247
H	3.894349	-3.043511	1.446019
H	2.263265	-2.38619	1.616212
C	3.642437	-1.056898	0.628602
H	3.901338	-0.538215	1.557565
H	4.568863	-1.120555	0.025341
Li	1.178687	0.916479	0.609861
Li	-1.441542	0.79887	0.58686

3.2 Design of Experiment

Table S6: Selected model for design of experiment.

Design	Model	Total runs	Design runs	DF	Power	I-optimality	Condition number
Reduced Combinatorial	Quadratic	27	24-+	10	76	18.27	8.82

Table S7: Design summary.

**Objective**	**Optimization (RSM)**
Process model	Quadratic
Mixture model	--
**Design**	**Reduced Combinatorial**
Runs in design	24
Center points	0
Replicated runs	3
Replicates	0
N = actual runs	27
Maximum runs	12000
Constraints	No

Table S8: Descriptive Statistics.

	**Yield**
**Worksheet statistics**	
Worksheet runs	25
N	25
Min	0
Max	56
Mean	19,56
Q(25%)	8
Q(75%)	28
Median	19
Std. dev.	15,4356
Min/Max	0
Std. dev./Mean	0,789139
Skewness	0,431481
Skewness test	0,93055
Kurtosis	-0,180341
**Model statistics**	
Model type	Evaluation of PLS model
Scaling type	All factors are scaled to unit variance
DF	19
R2	0,861465
R2 adj	0,825009
Q2	0,725616
Condition number	1,30996
Model terms	6
DF residual	19
RSD	6,457
p model	1,51629e-07
DF lack of fit	16
p lack of fit	0,0946856
DF pure error (repl. runs)	3
SD pure error	2,97209
Residual skewness	-0,264076
Residual skewness test	-0,569517

Table S9: Summary of Fit List.

	**R2**	**R2 Adj.**	**Q2**	**SDY**	**RSD**	**N**	**Model Validity**	**Reproducibility**
Yield	0,861465	0,825009	0,725616	15,4356	6,457	25	0,409858	0,962925
N = 25	Cond. no. =	1,31						
Comp. = 2								

Table S10: ANOVA Table.

**Yield**	**DF**	**SS**	**MS (variance)**	**F**	**p**	**SD**
Total	25	15283	611,32			
Constant	1	9564,84	9564,84			
Total corrected	24	5718,16	238,257			15,4356
Regression	5	4925,99	985,199	23,6299	**0,000**	31,3879
Residual	19	792,165	41,6929			6,457
Lack of Fit (Model error)	16	765,665	47,854	5,41744	**0,095**	6,91766
Pure error (Replicate error)	3	26,5	8,83333			2,97209
	N = 25	Q2 = 0,726		Cond. no. = 1,31		
	DF = 19	R2 = 0,861		RSD = 6,457		
	Comp. = 2	R2 adj. = 0,825				

Table S11: Worksheet for Design of Experiment (reaction setup = 0 is open; reaction setup = 1 is closed).

Exp No	Exp Name	Run Order	Incl/Excl	Time [min]	Temperature [°C]	Equivalents Amine	Reaction setup	Yield [%]
1	N1	26	Incl	60	90	1	0	0
2	N2	22	Excl	120	100	1	1	8
3	N3	3	Incl	5	120	1	0	0
4	N4	15	Incl	5	90	1	1	25
5	N5	7	Incl	5	100	1	1	15
6	N6	21	Incl	120	120	1	0	0
7	N7	14	Incl	60	90	1	1	32
8	N8	16	Excl	60	100	1	0	20
9	N9	25	Incl	60	120	2	0	0
10	N10	1	Incl	120	90	2	0	8
11	N11	6	Incl	60	100	2	1	41
12	N12	4	Incl	5	120	2	1	14
13	N13	19	Incl	5	90	2	0	10
14	N14	11	Incl	5	100	2	0	0
15	N15	5	Incl	120	120	2	1	46
16	N16	2	Incl	120	90	2	1	28
17	N17	13	Incl	120	100	5	1	56
18	N18	24	Incl	5	120	5	1	28
19	N19	23	Incl	60	90	5	1	26
20	N20	20	Incl	5	100	5	0	15
21	N21	17	Incl	60	120	5	1	31
22	N22	8	Incl	120	90	5	0	17
23	N23	10	Incl	60	100	5	0	19
24	N24	18	Incl	120	120	5	0	28
25	N25	27	Incl	120	120	1	0	0
26	N26	9	Incl	5	120	5	1	26
27	N27	12	Incl	120	90	5	0	24

As factors for the design of experiment time (5 min, 60 min, 120 min), temperature (90 °C, 100 °C, 120 °C), equivalents of amine (1 eq., 2 eq., 5 eq.) and reaction setup (0 = open; 1 = closed) were chosen. The time slot up to 2 hours was chosen due to pre-experiments, where 2 hours seemed to display the best compromise between long reaction time and little *t*-butyl lithium decomposition, as discussed on page 4 of this Supporting Information. As response yield [%] was chosen. All experiments were carried out as described in 2. Experimental section. The yields were determined by ^{1}H-NMR spectroscopy by comparison of the integral I_3 at 1.52 ppm of *N*-methylpiperidine (**1**) which accounts for 4 protons and the integral I_7 at 3.04 ppm of product **7** which accounts for 2 protons, and referenced to the stoichiometry of amine to *t*-butyllithium.

$$Y[\%] = \frac{\frac{I_7}{2} \times eq_{Amine}}{\frac{I_3}{4} + \frac{I_7}{2}} \times 100 \qquad (1)$$

For the chosen electrophile benzophenone, neither decomposition products of *t*-butyllithium nor the addition product of *t*-butyllithium to benzophenone itself were observed in the NMR spectra. However, if using *tri*-methylchlorosilane as electrophile for the lithiated amine **2**, the trapping product of *t*-butyllithium **9** can be observed in the ^{29}Si-NMR spectrum as shown in Figure S20. Therefore, we assume that the observed yields can be explained by remaining *t*-butyllithium that does not react with benzophenone under the given conditions. The use of *tri*-methylchlorosilane as electrophile did not seem useful as possible hydrochlorides of amine **1** or the addition product **9** may be formed in the reaction which makes the determination of the yield by NMR not applicable. Therefore, the reaction with *tri*-methylchlorosilane has not been quantified. To assure a homogeneous reaction solution without formation of solids, benzophenone as trapping reagent was chosen.

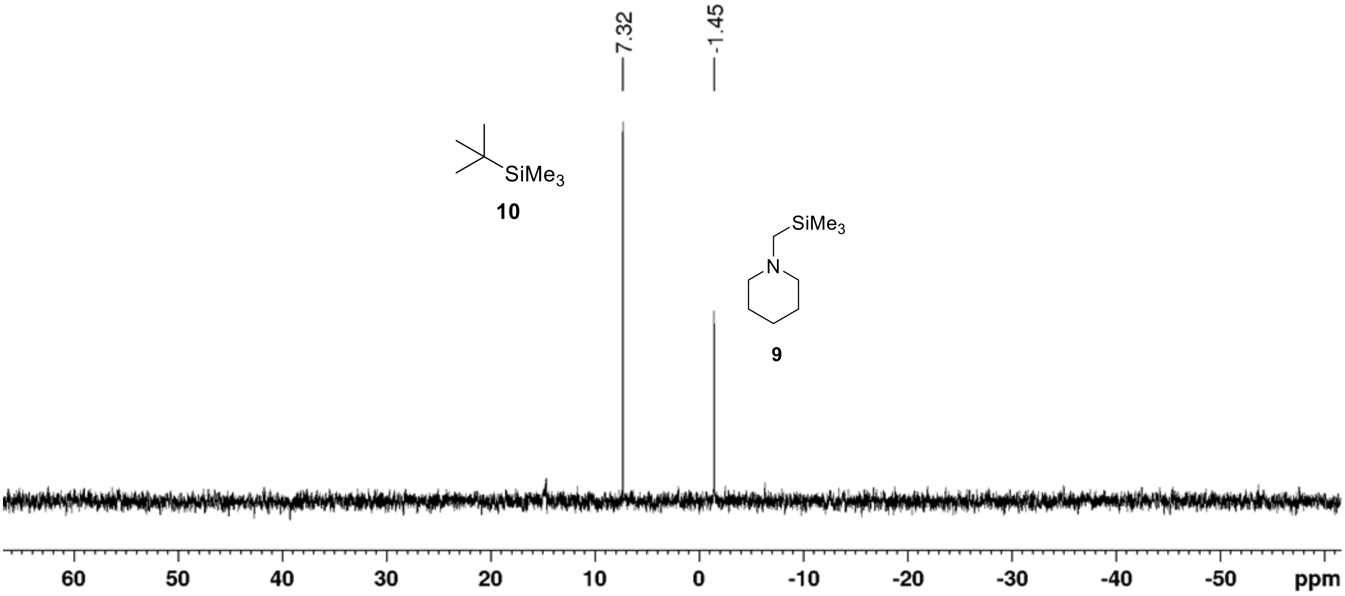

Figure S20: ^{29}Si-NMR of deprotonation of **1** under microwave conditions and trapping with *tri*-methylchlorosilane.

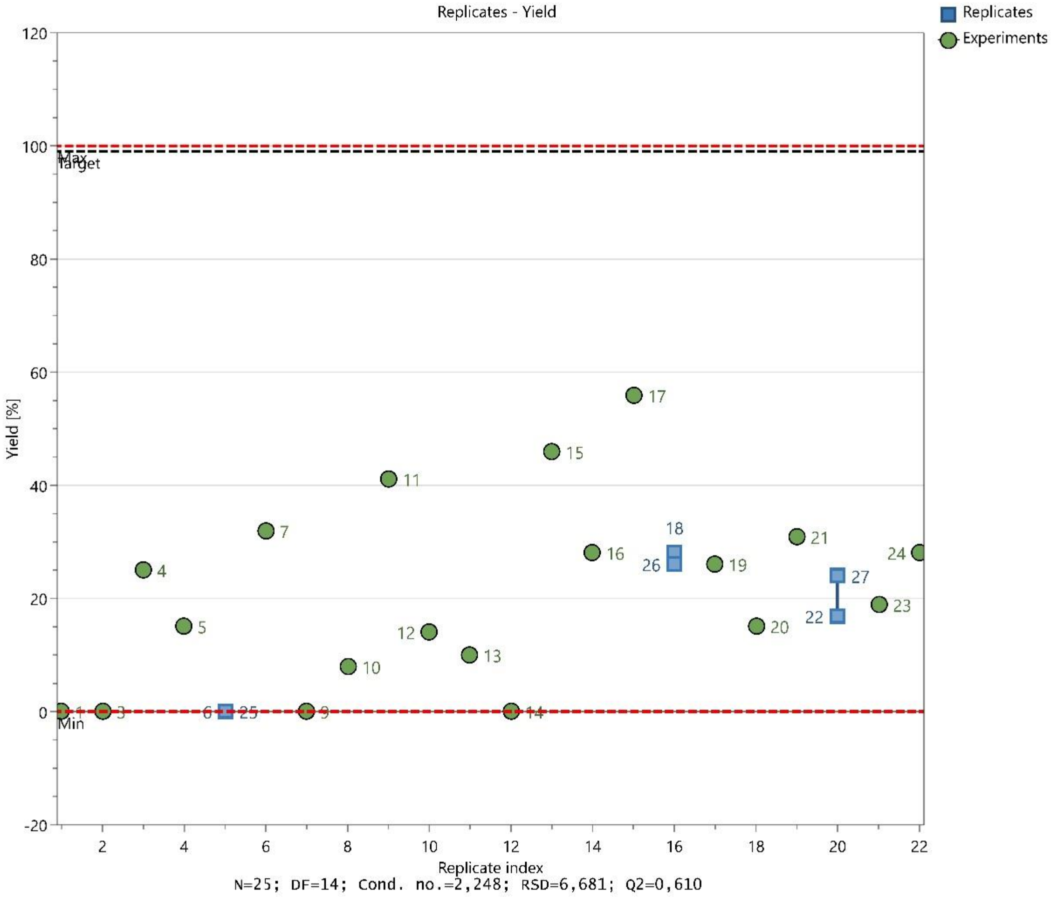

Figure S21: Replicate plot for DoE of direct deprotonation of *N*-methylpiperidine under microwave conditions.

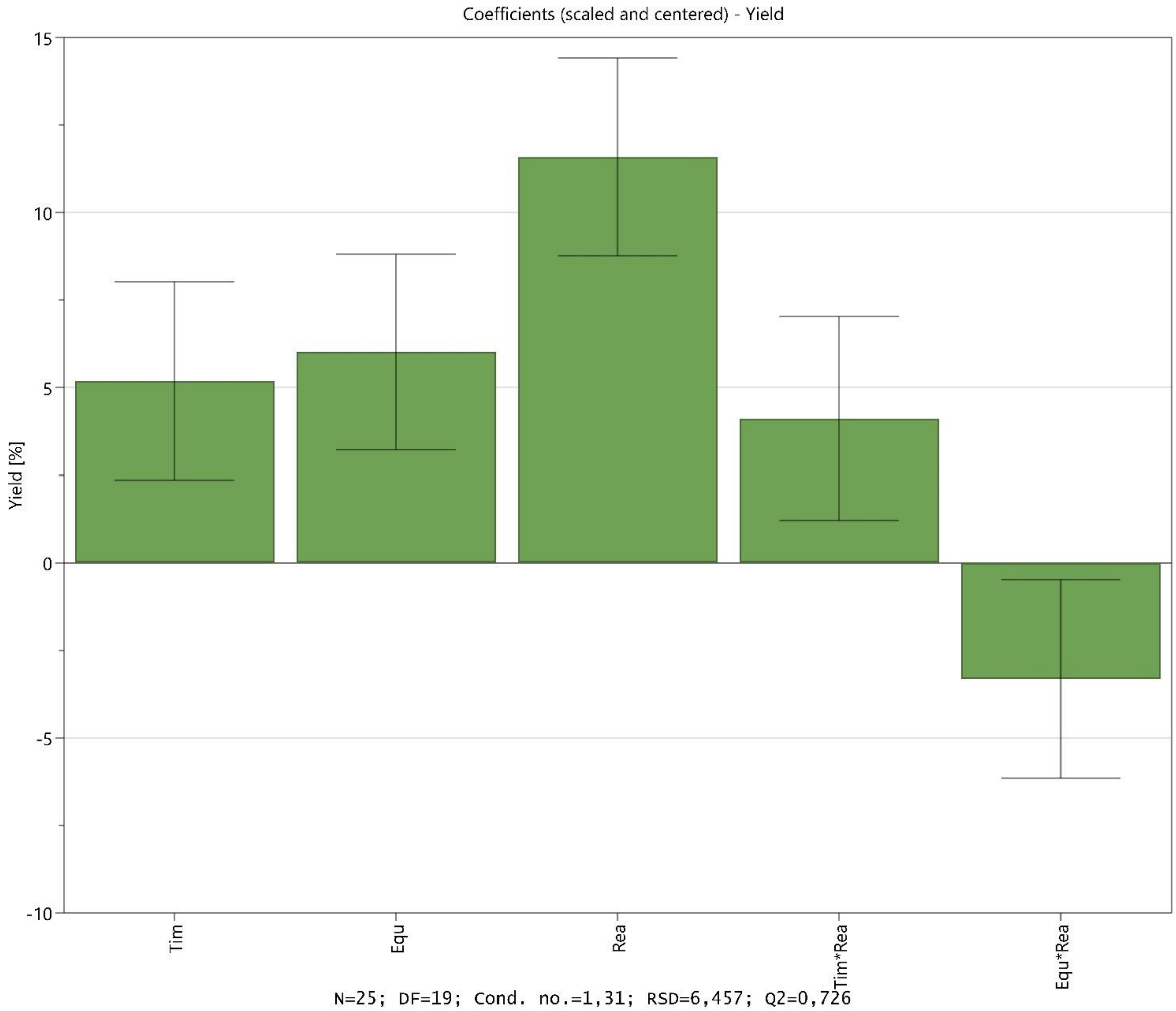

Figure S22: Coefficients plot for DoE of direct deprotonation of *N*-methylpiperidine under microwave conditions.

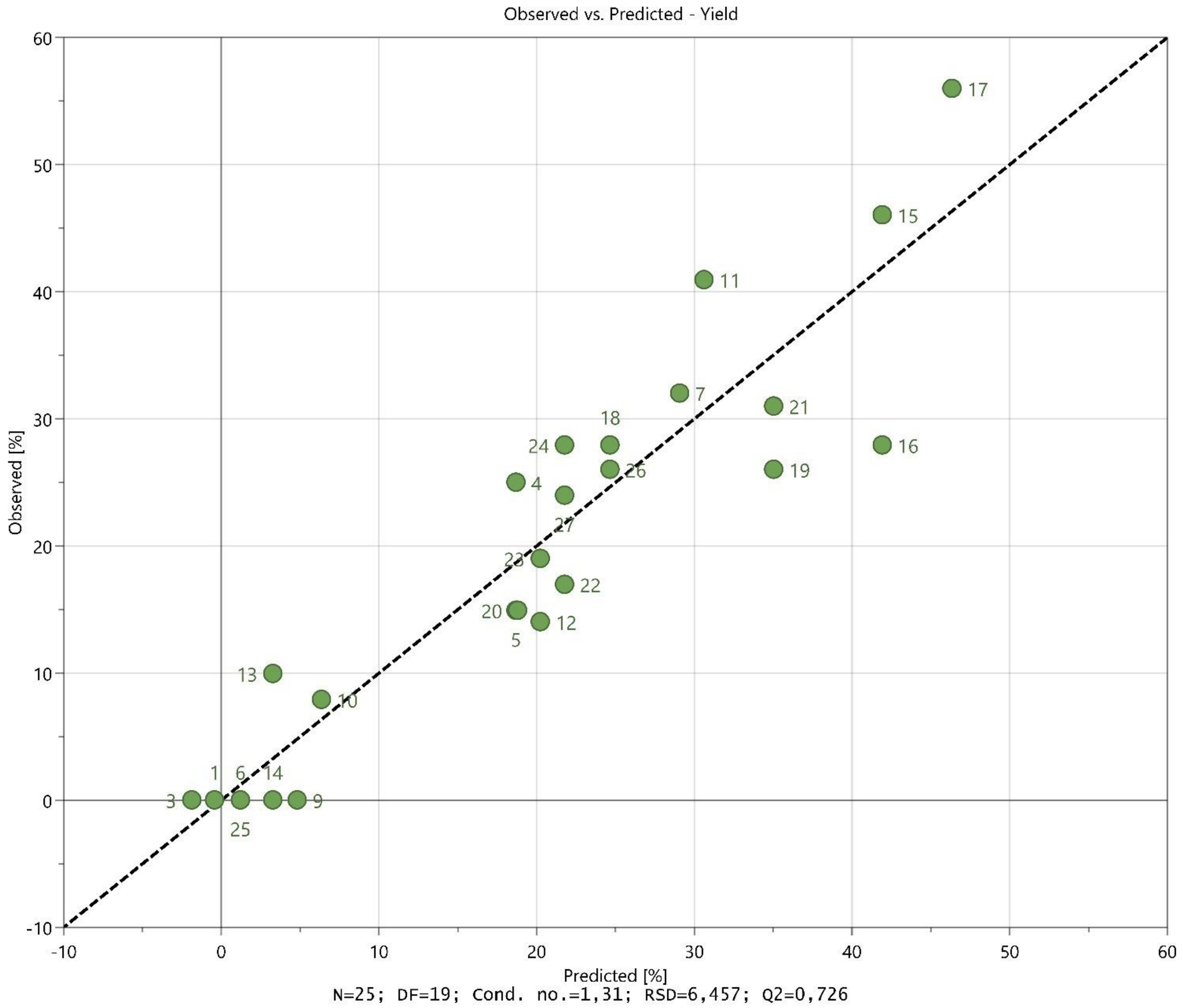

Figure S23: Observed vs. Predicted plot for DoE of direct deprotonation of *N*-methylpiperidine under microwave conditions.

The NMR spectra for each experiment are shown below.

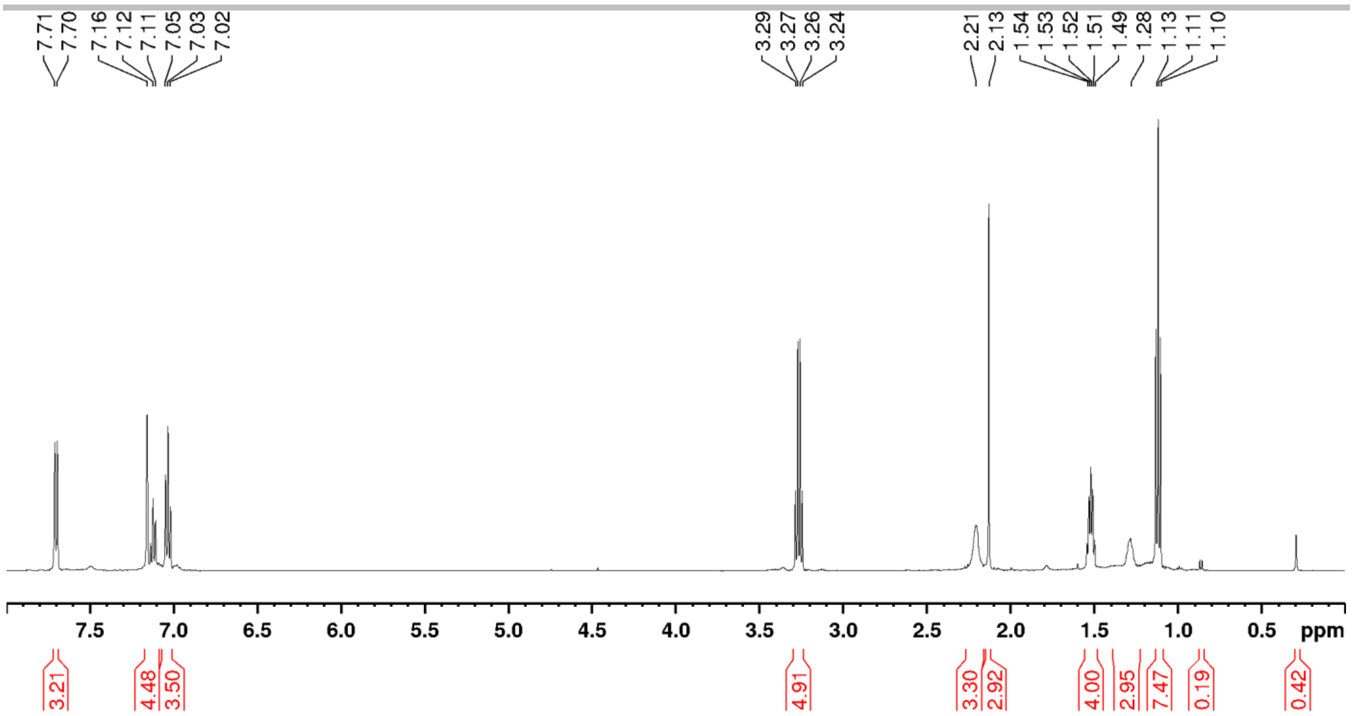

Figure S24: ^{1}H-NMR spectrum of reaction N1.

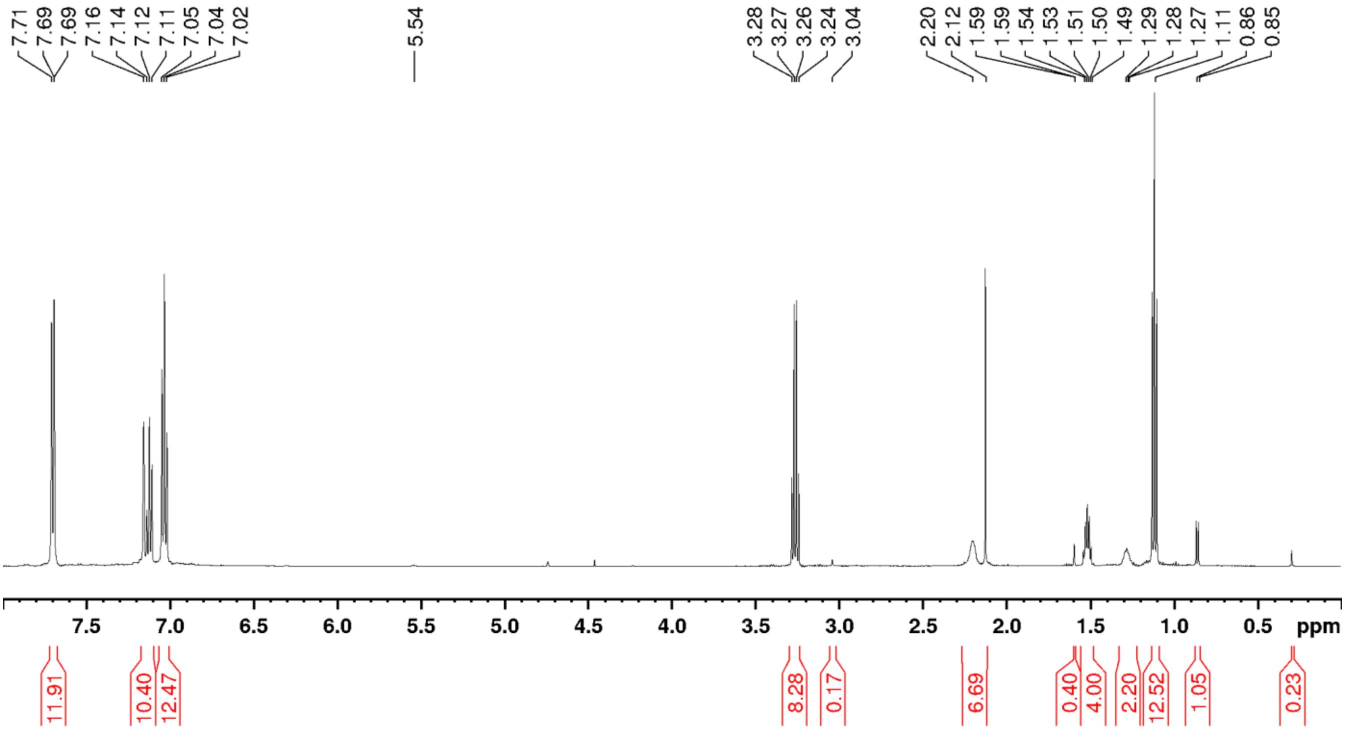

Figure S25: ^{1}H-NMR spectrum of reaction N2.

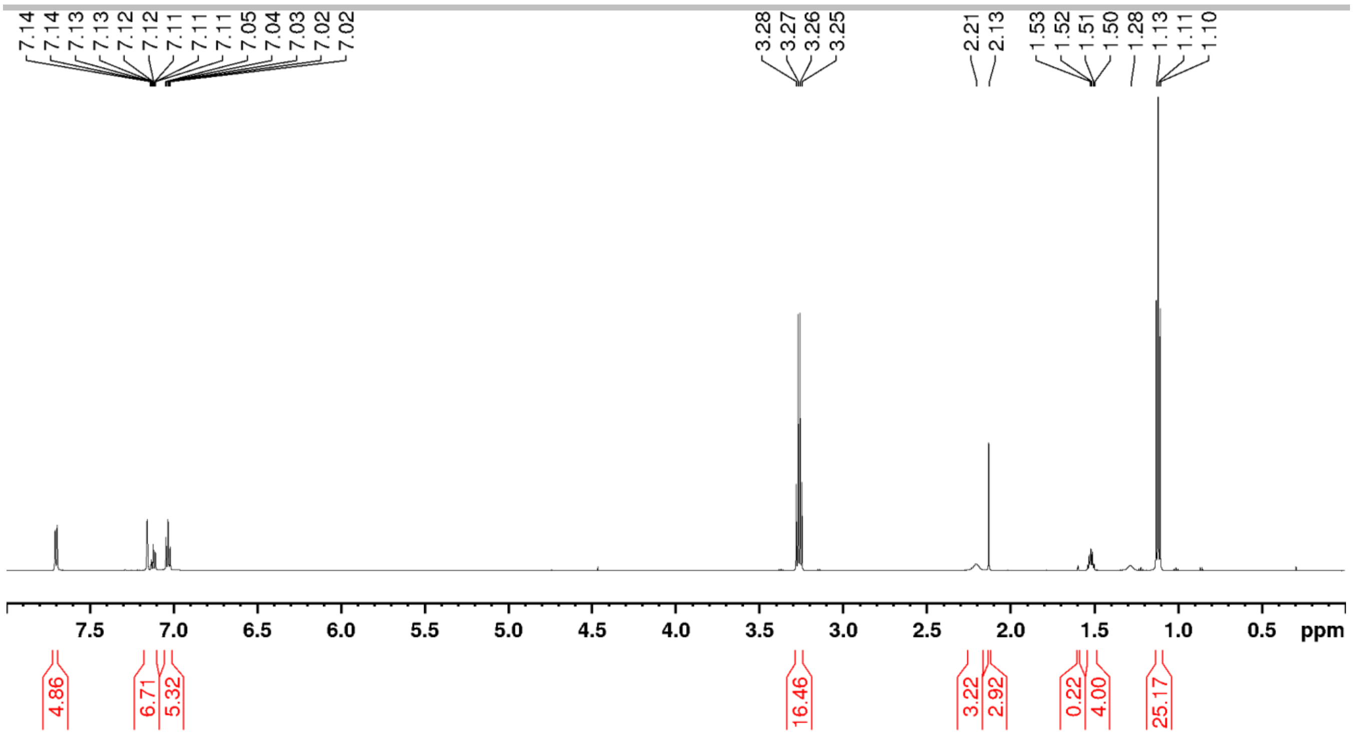

Figure S26: ^{1}H-NMR spectrum of reaction N3.

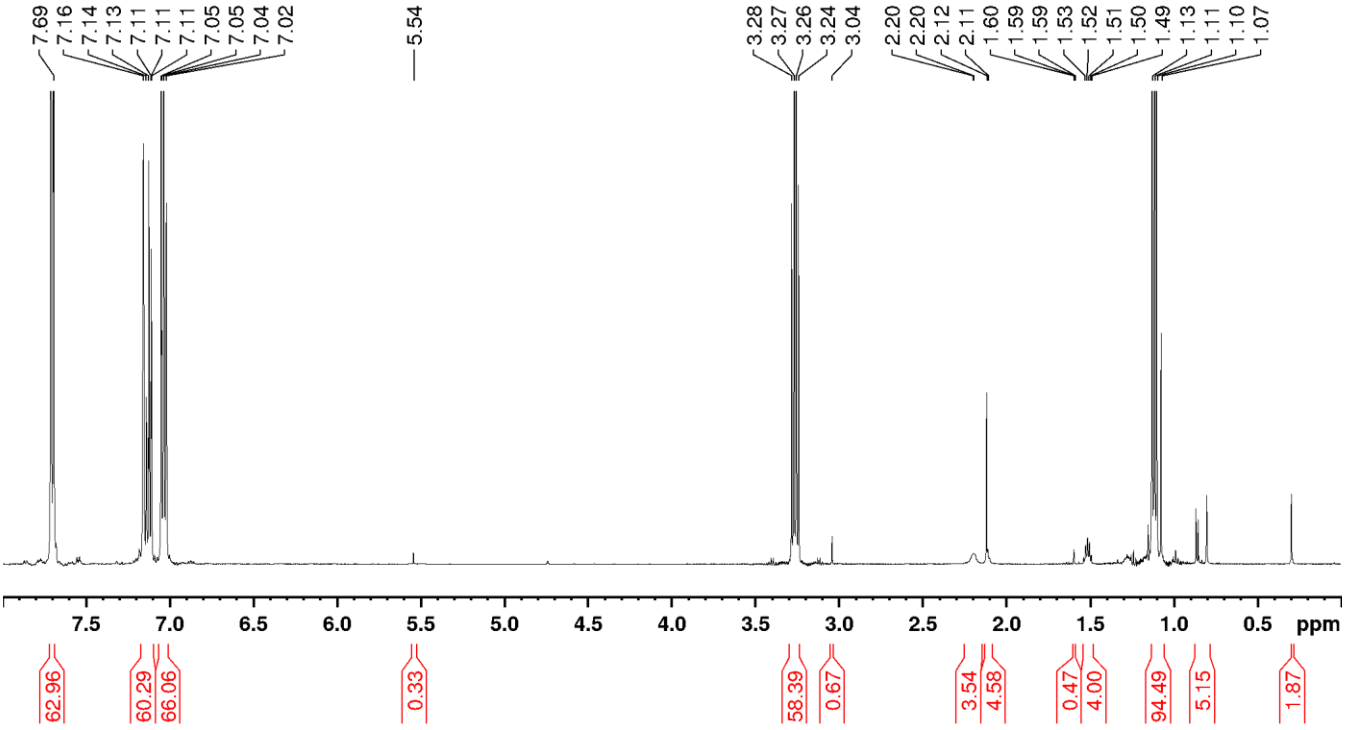

Figure S27: ^{1}H-NMR spectrum of reaction N4.

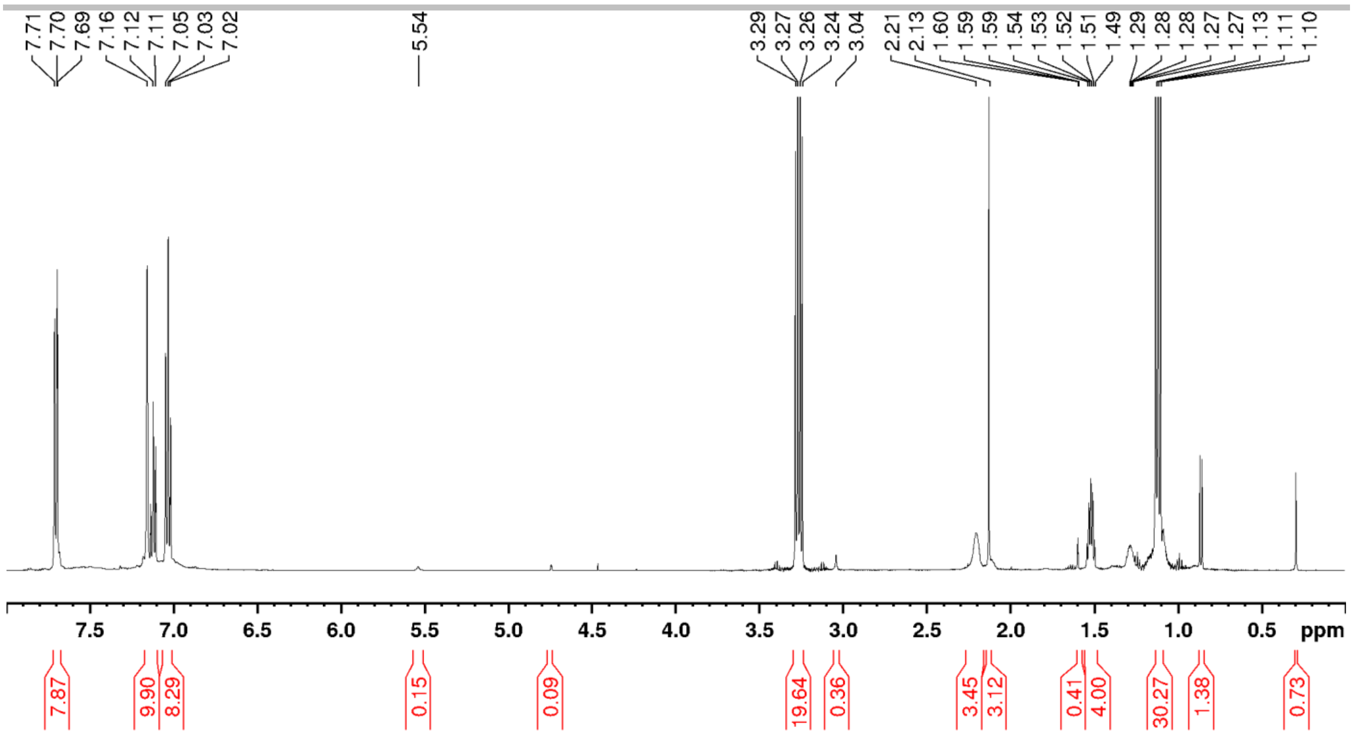

Figure S28: ^{1}H-NMR spectrum of reaction N5.

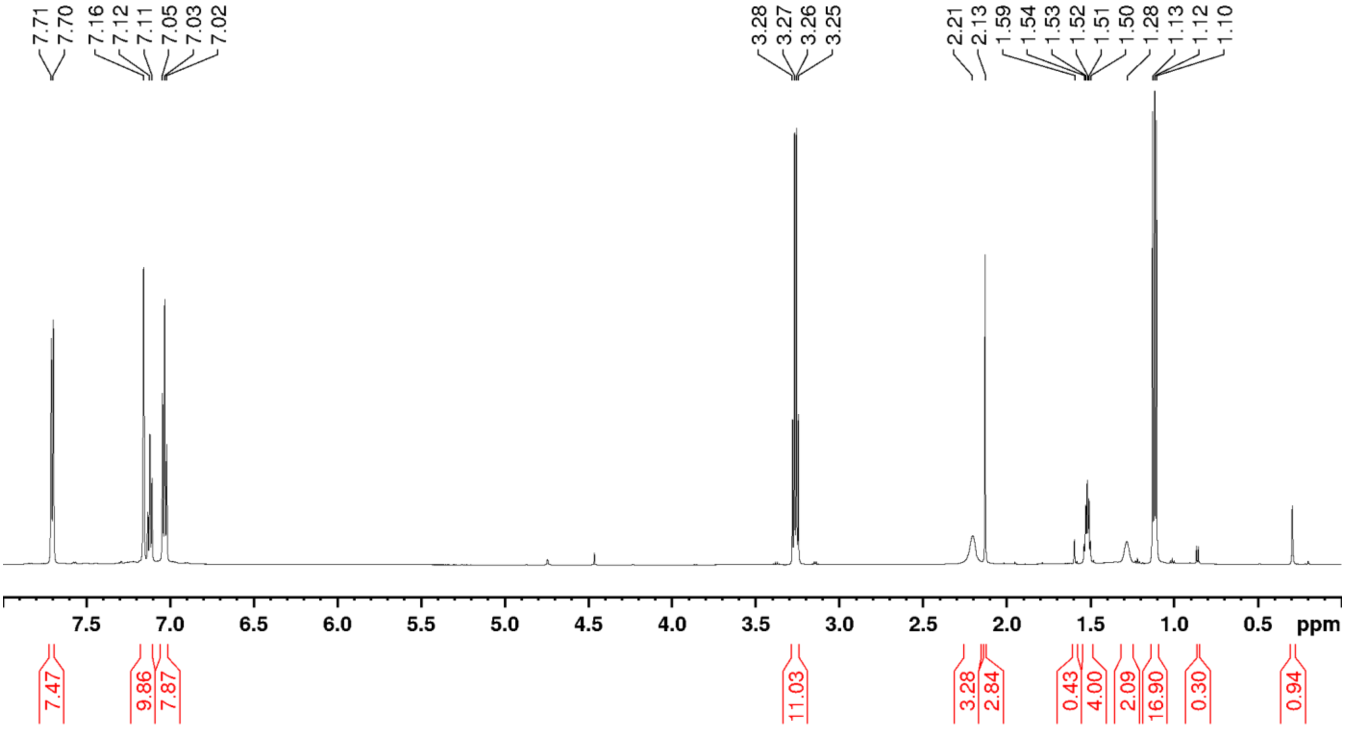

Figure S29: ^{1}H-NMR spectrum of reaction N6.

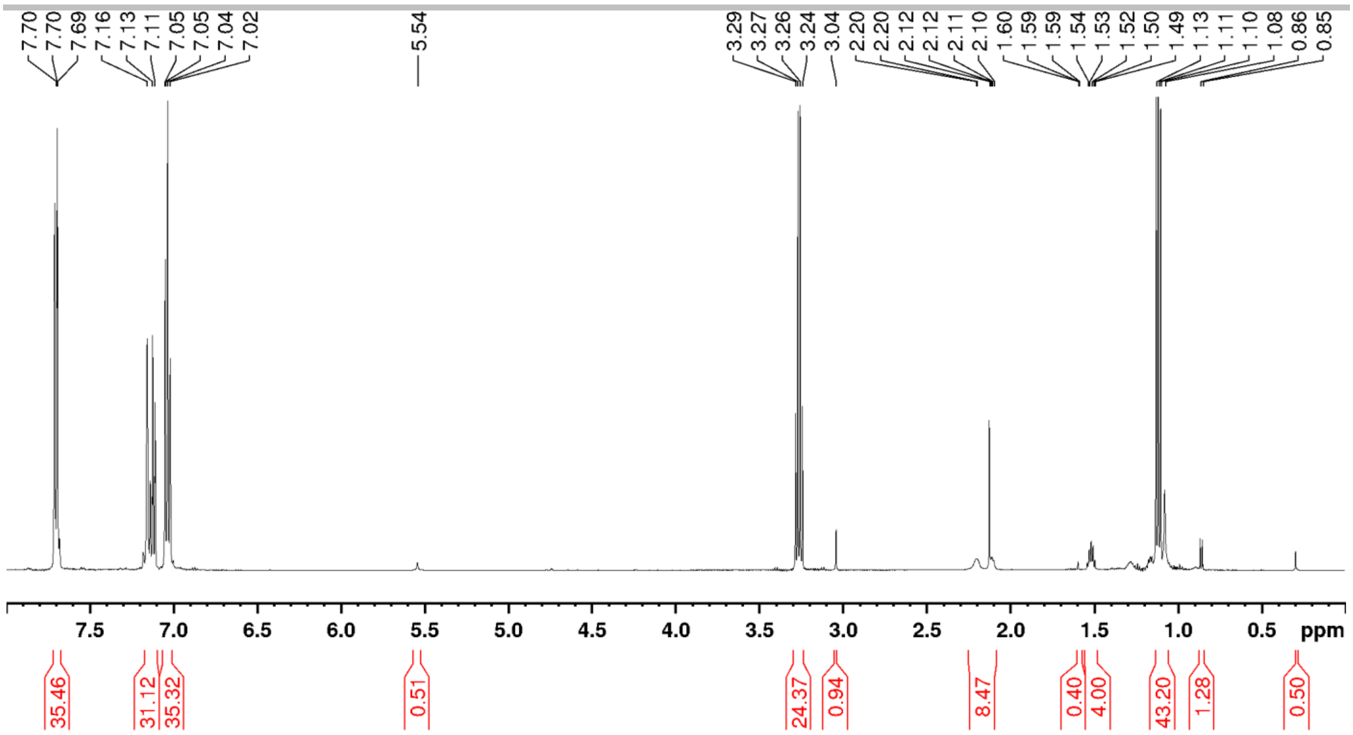

Figure S30: ^{1}H-NMR spectrum of reaction N7.

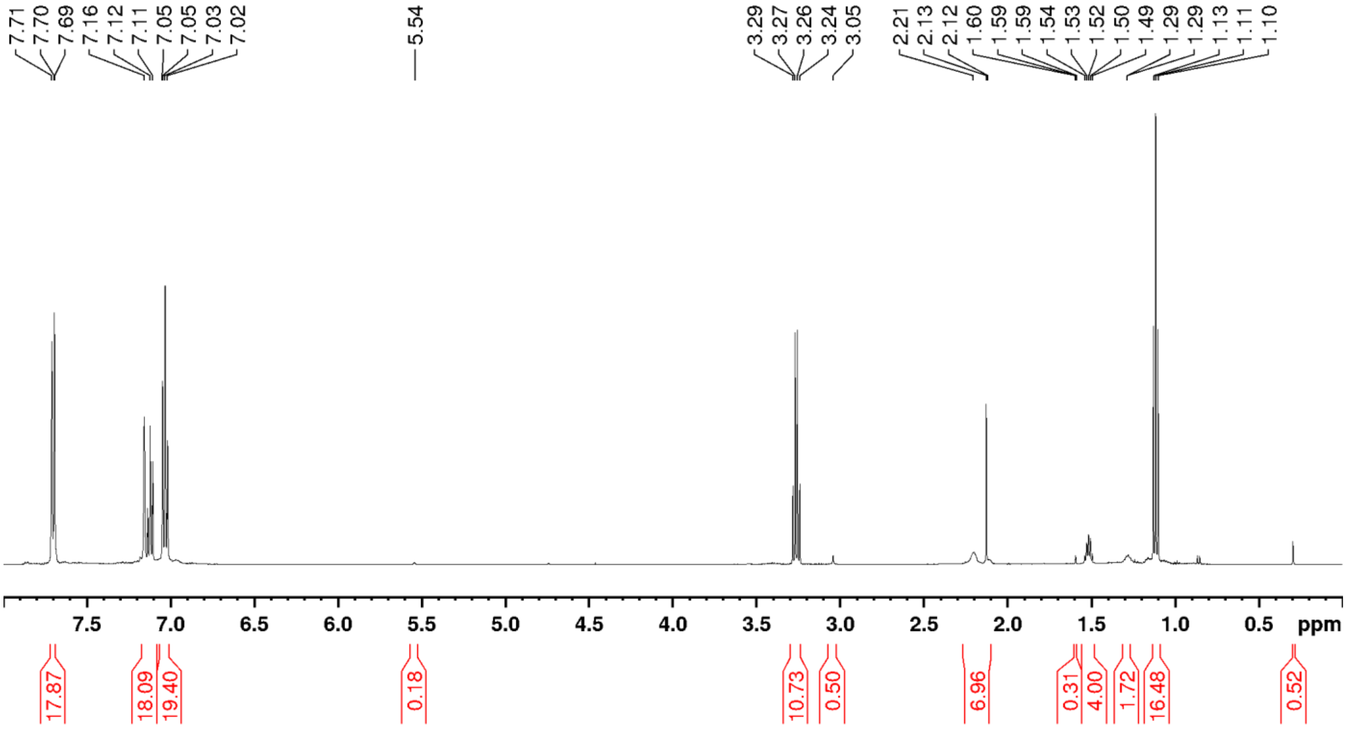

Figure S31: ^{1}H-NMR spectrum of reaction N8.

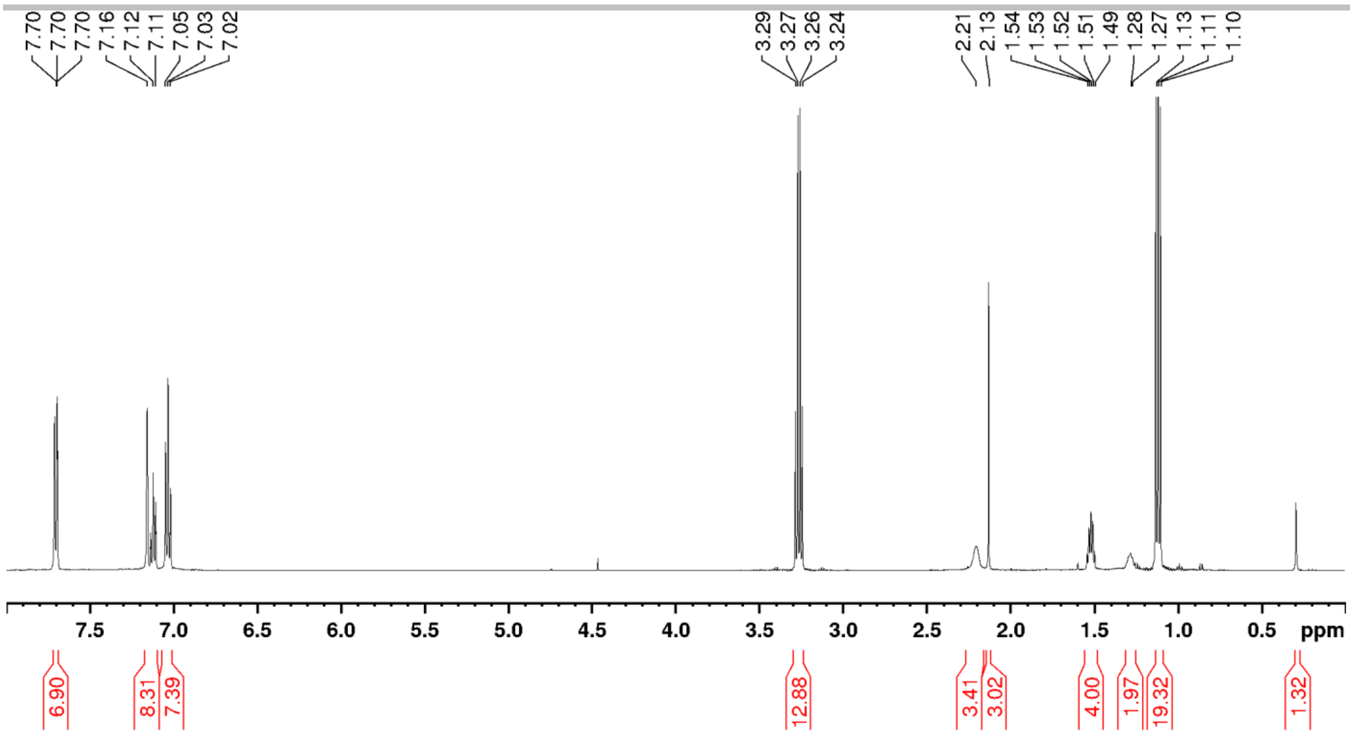

Figure S32: ^{1}H-NMR spectrum of reaction N9.

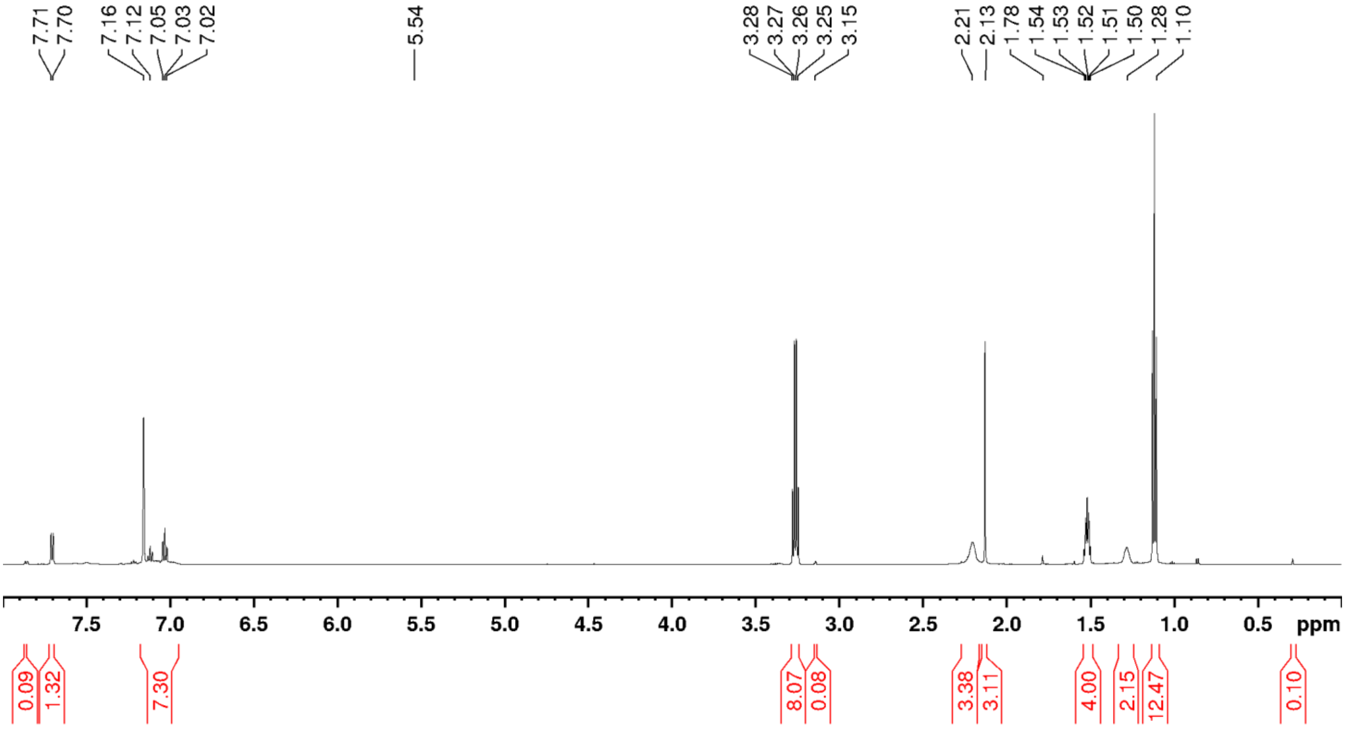

Figure S33: ^{1}H-NMR spectrum of reaction N10.

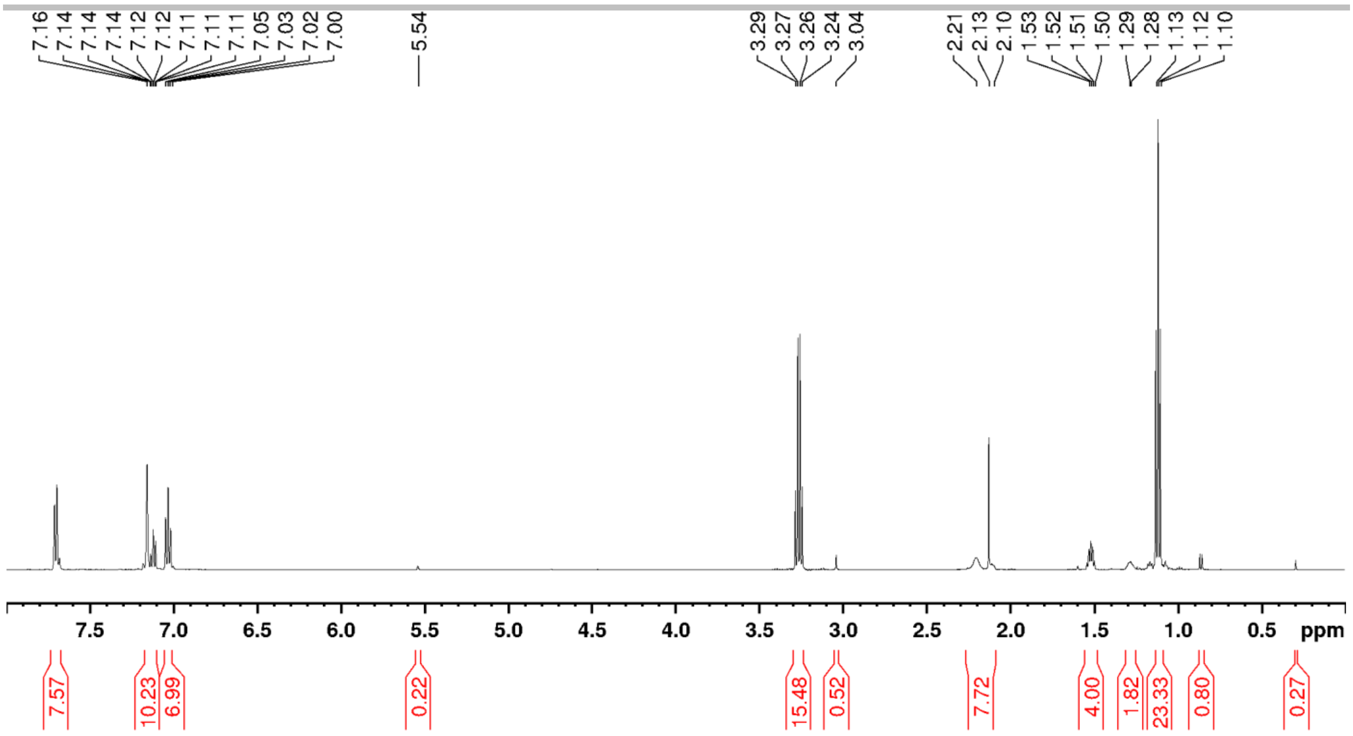

Figure S34: ^{1}H-NMR spectrum of reaction N11.

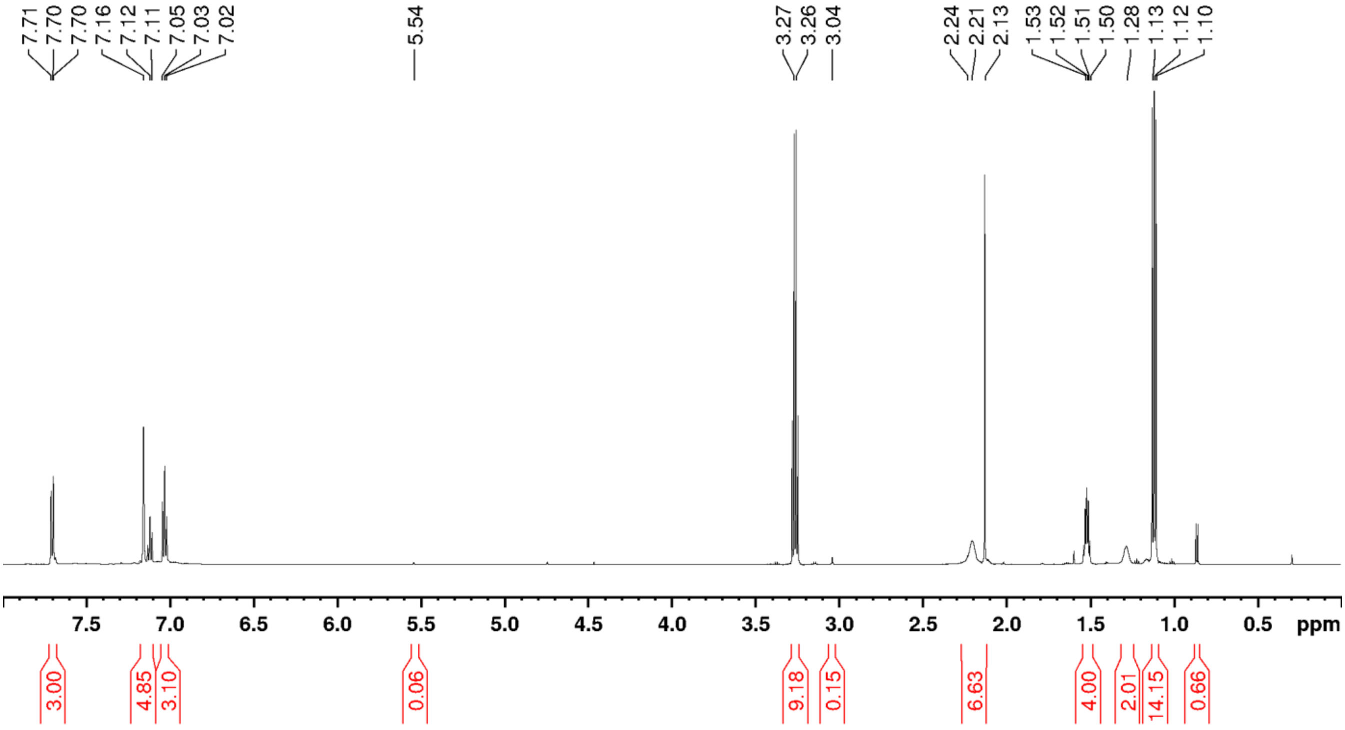

Figure S35: ^{1}H-NMR spectrum of reaction N12.

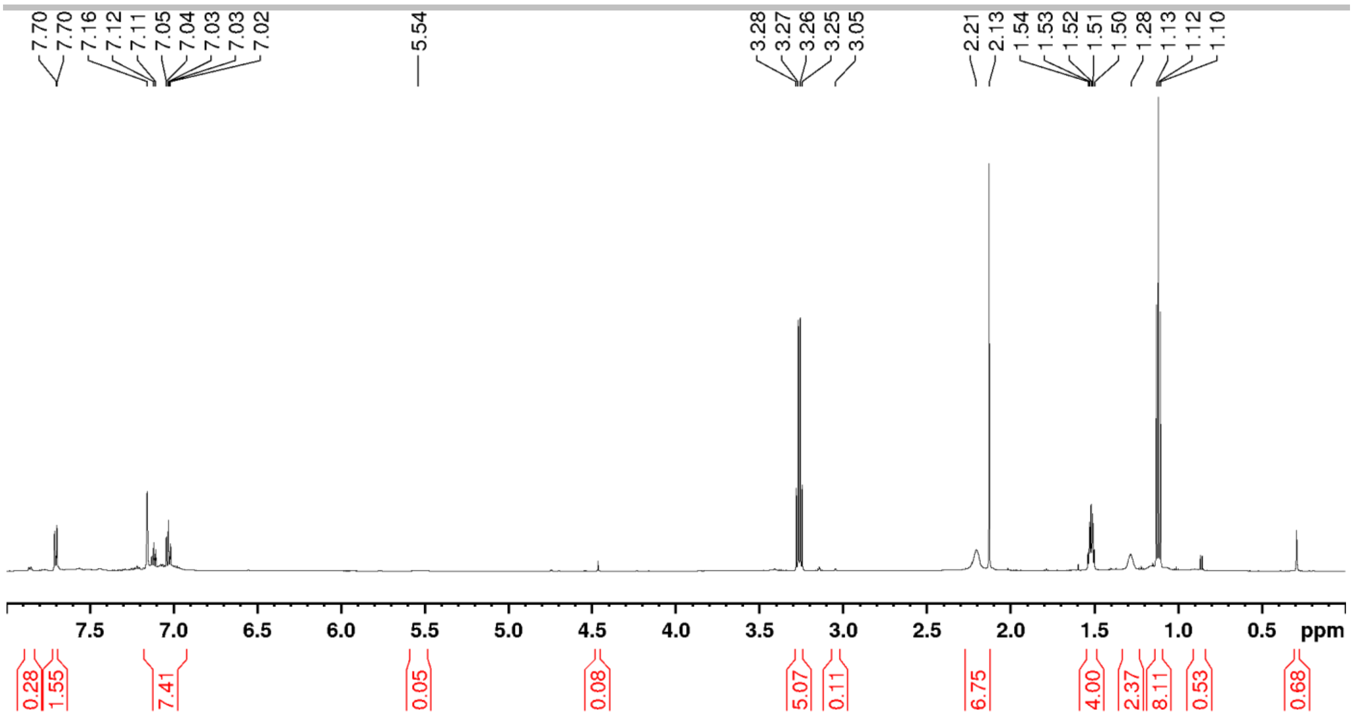

Figure S36: ^{1}H-NMR spectrum of reaction N13.

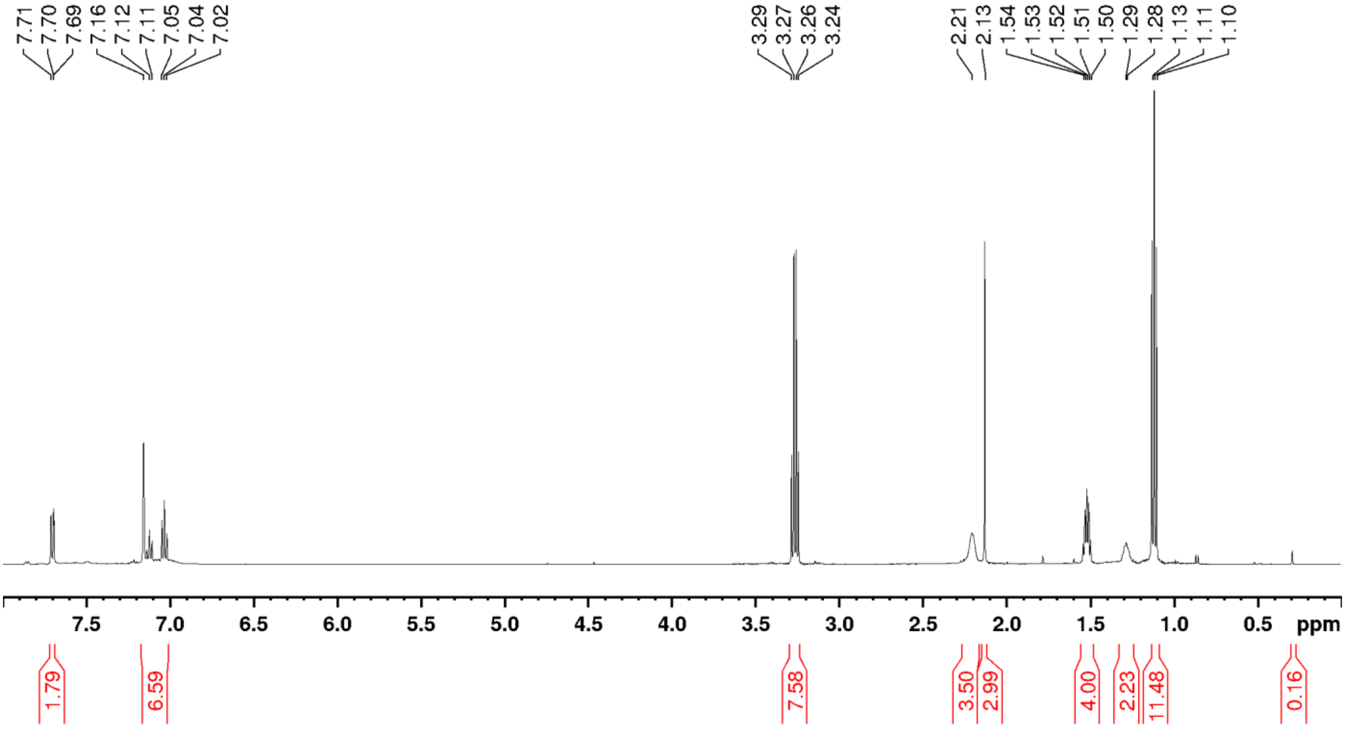

Figure S37: ^{1}H-NMR spectrum of reaction N14.

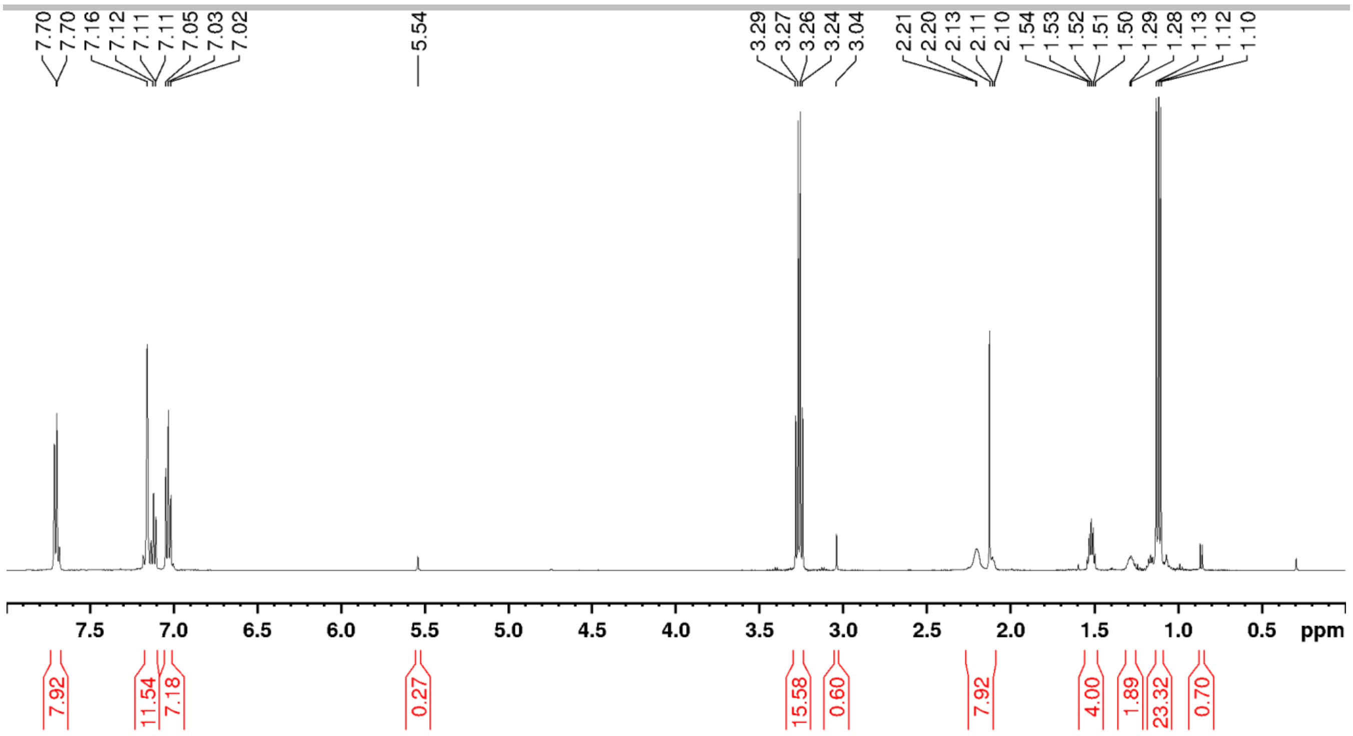

Figure S38: ^{1}H-NMR spectrum of reaction N15.

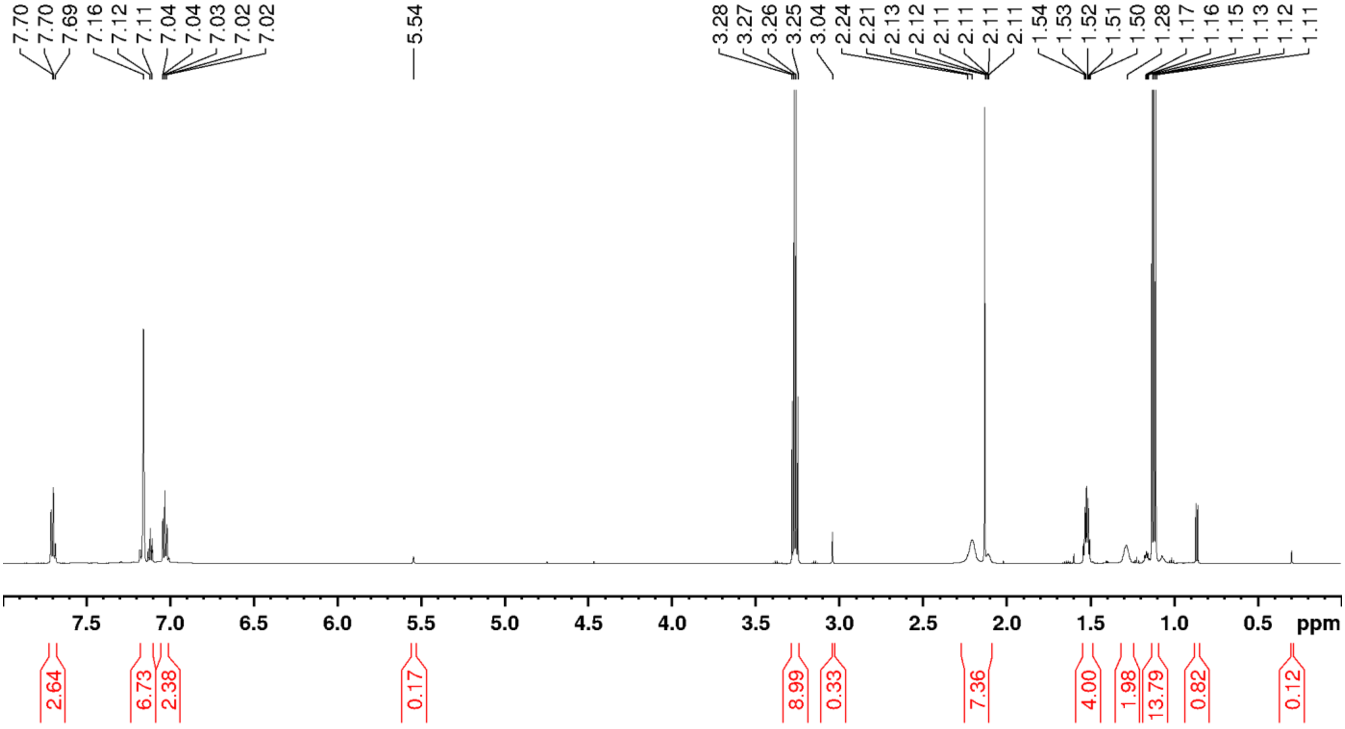

Figure S39: ^{1}H-NMR spectrum of reaction N16.

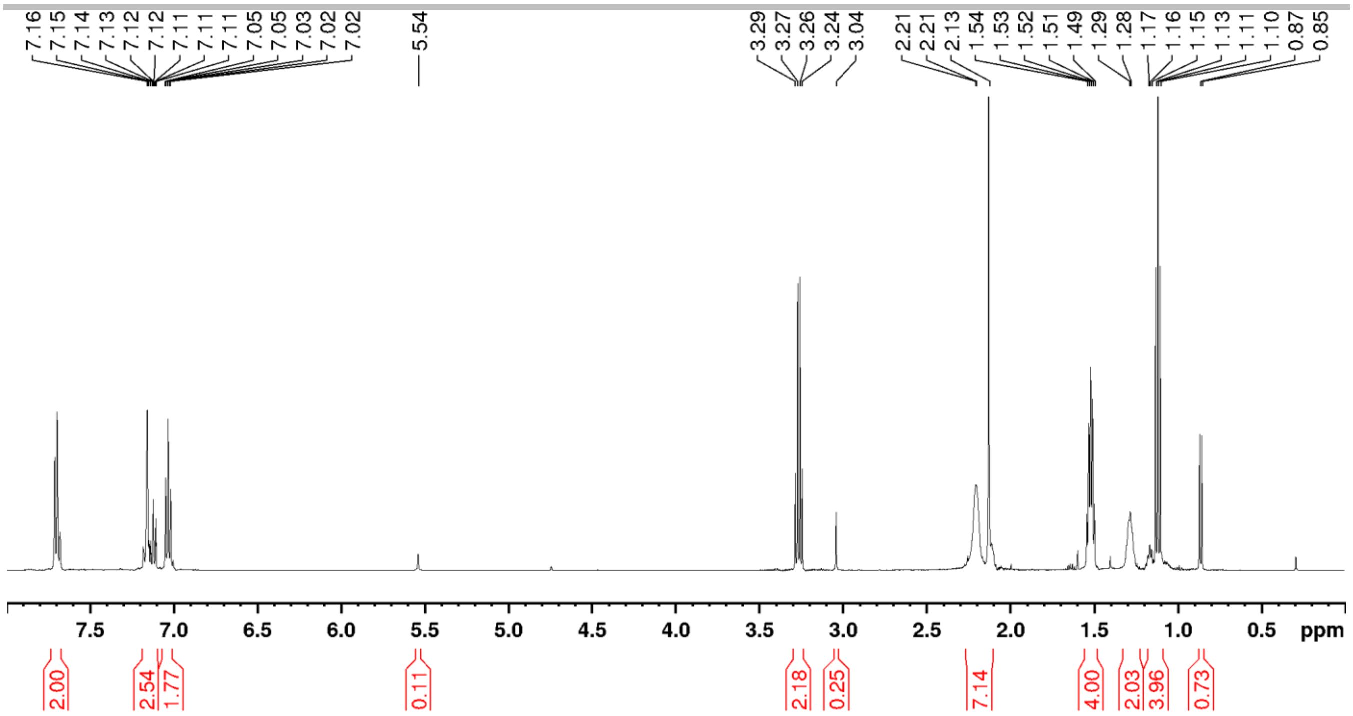

Figure S40: ^{1}H-NMR spectrum of reaction N17.

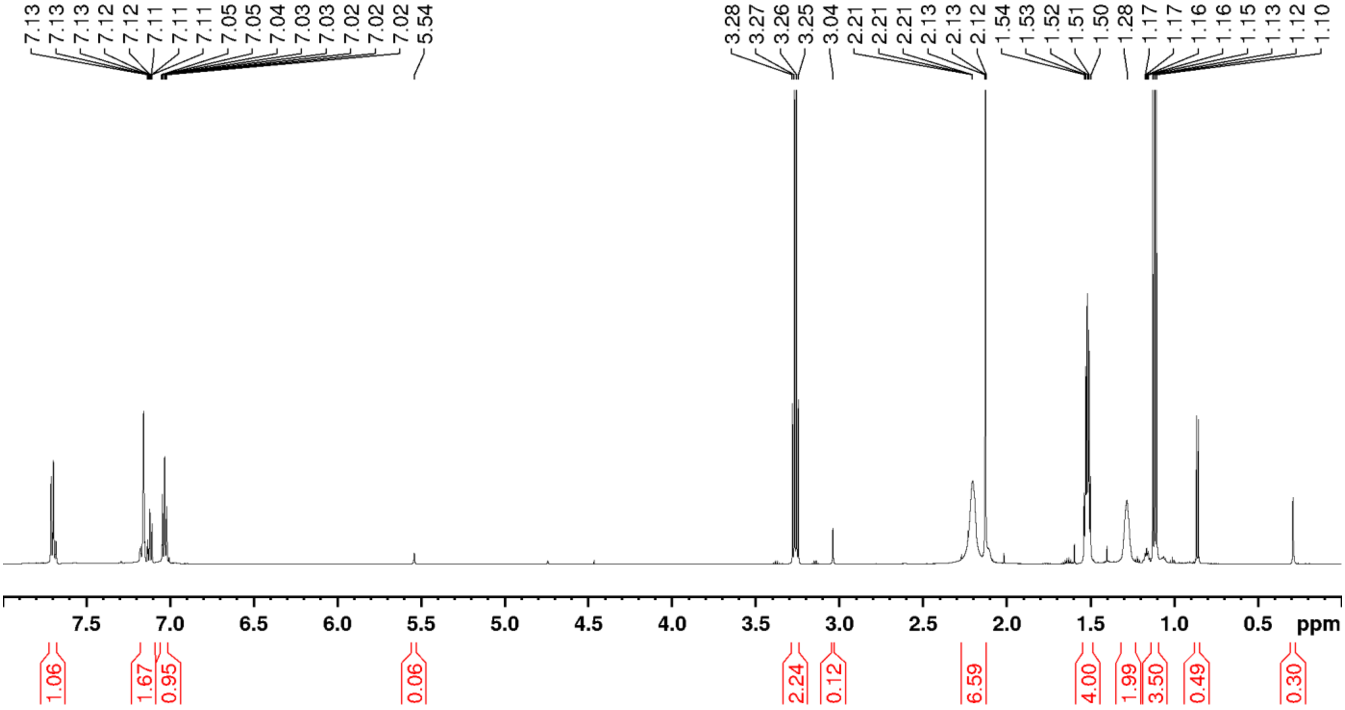

Figure S41: ^{1}H-NMR spectrum of reaction N18.

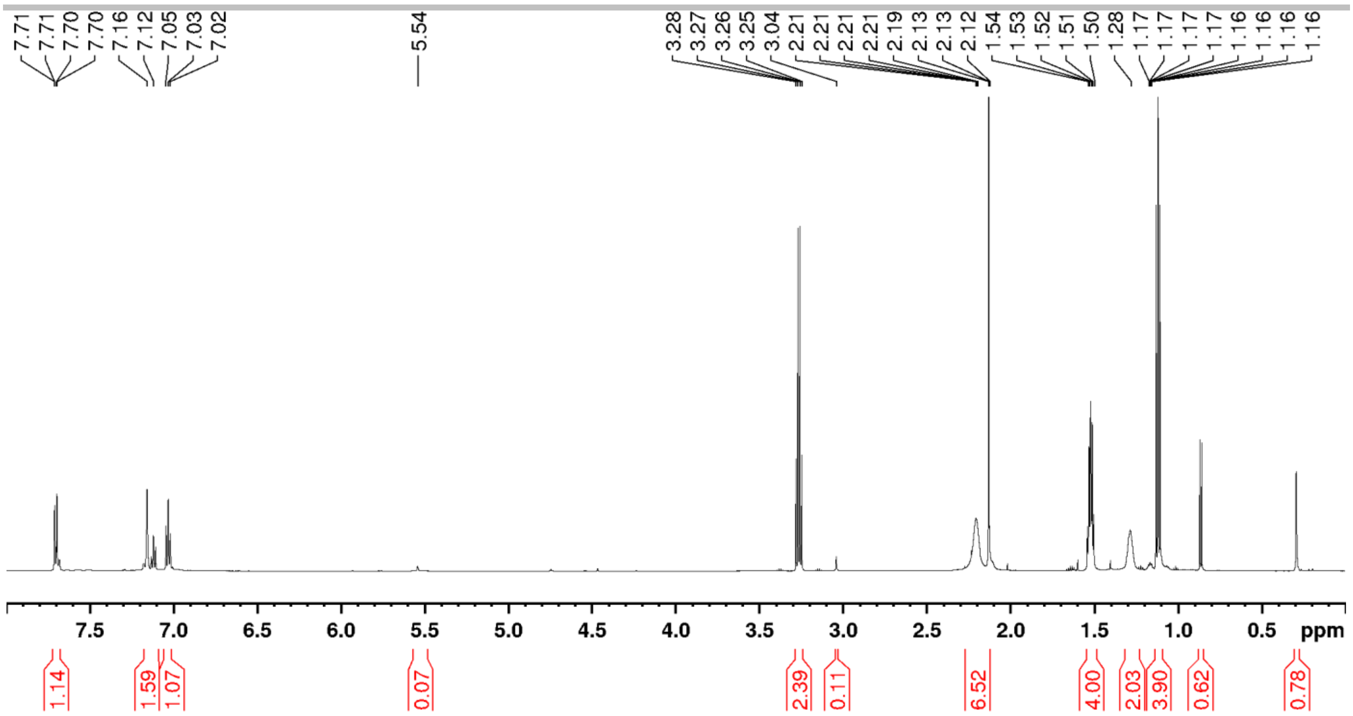

Figure S42: ^{1}H-NMR spectrum of reaction N19.

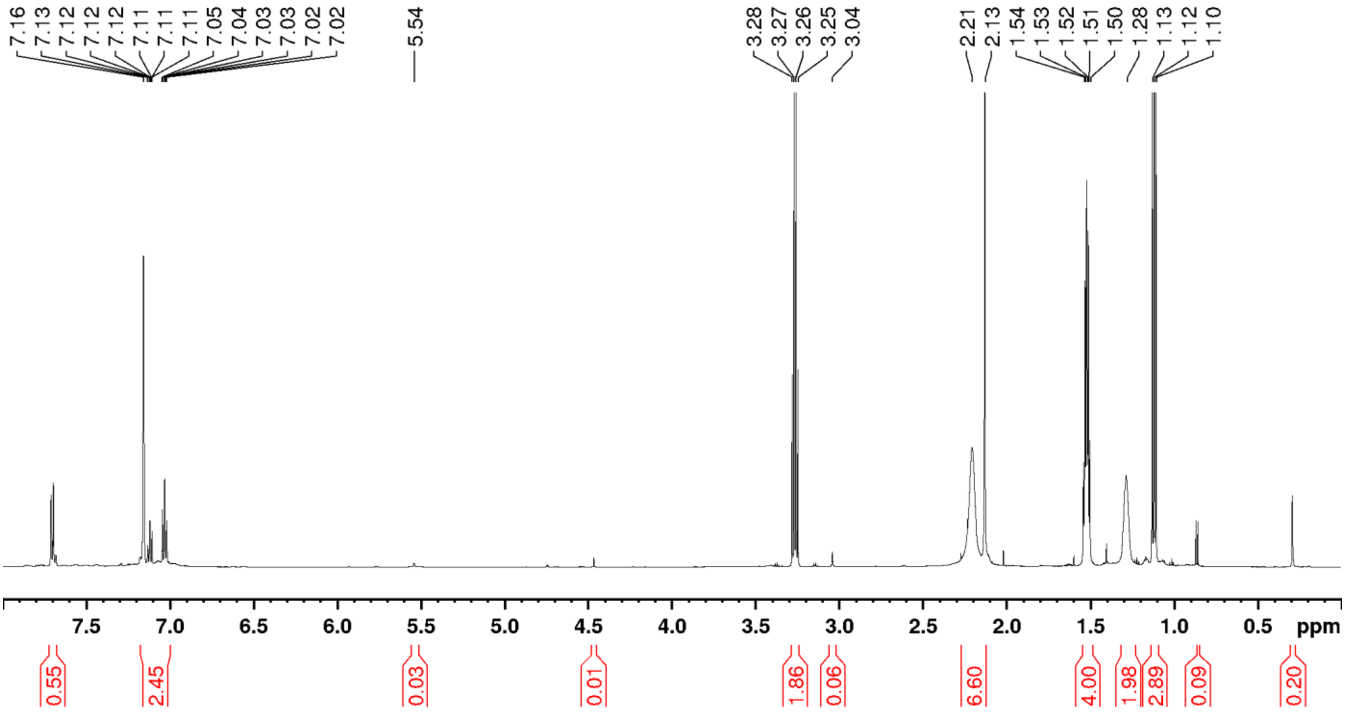

Figure S43: ^{1}H-NMR spectrum of reaction N20.

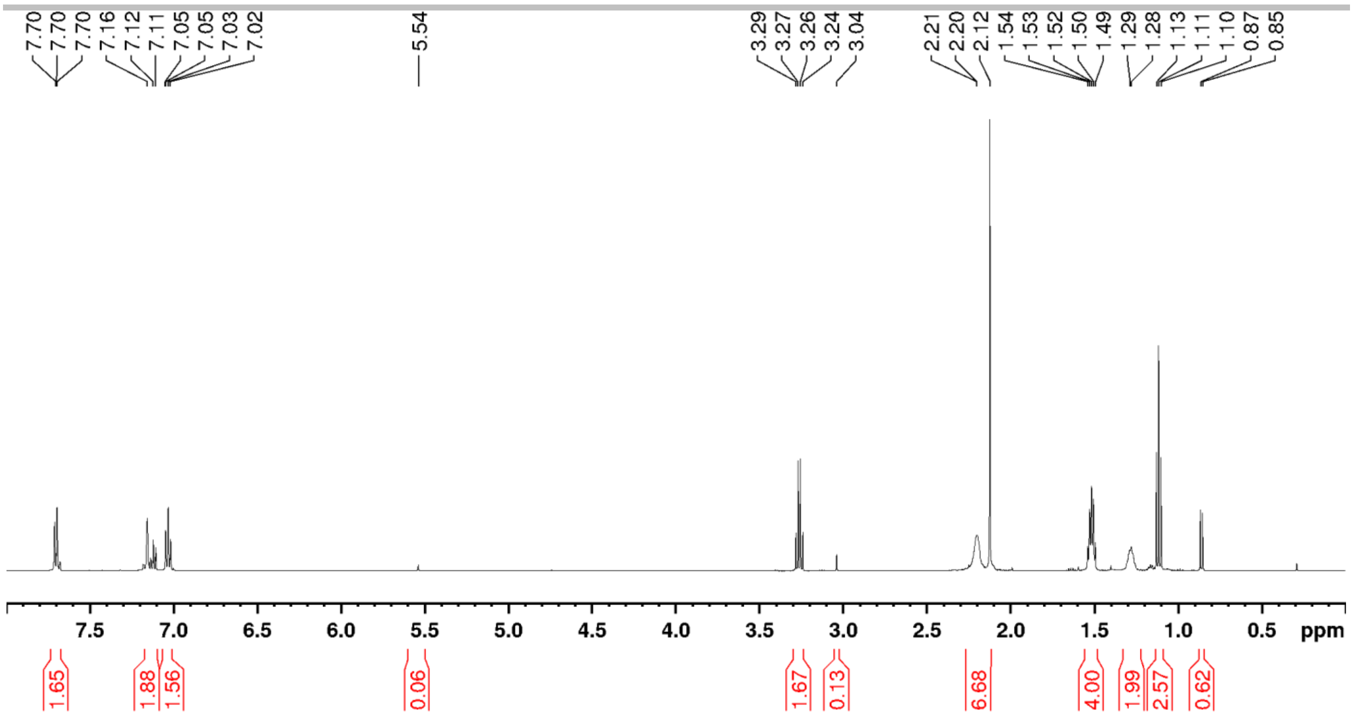

Figure S44: ^{1}H-NMR spectrum of reaction N21.

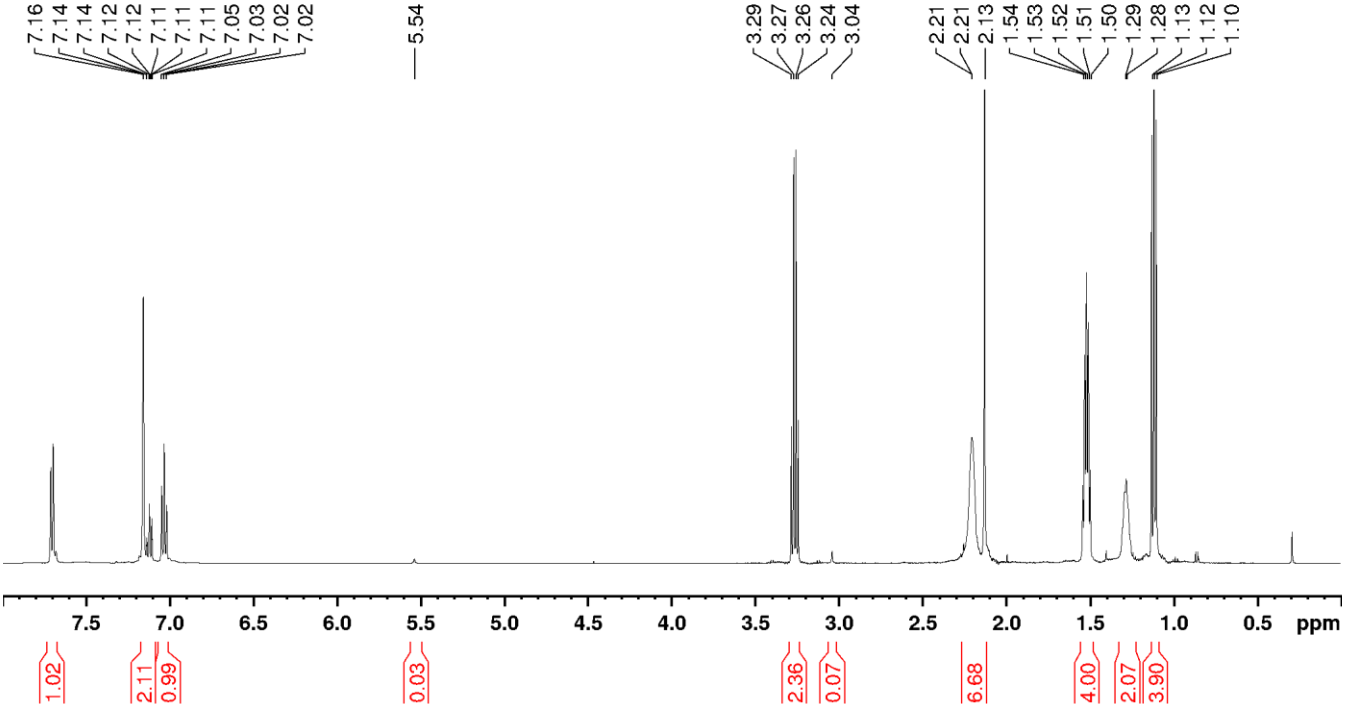

Figure S45: ^{1}H-NMR spectrum of reaction N22.

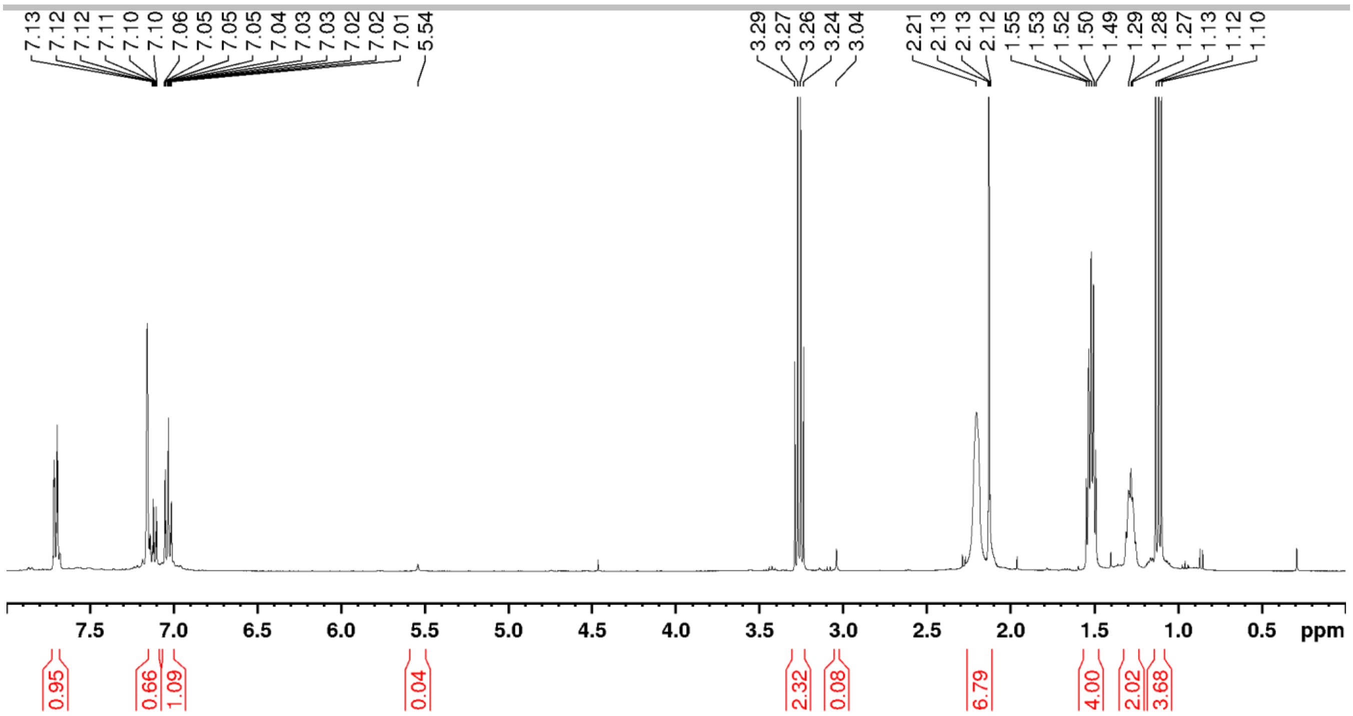

Figure S46: ^{1}H-NMR spectrum of reaction N23.

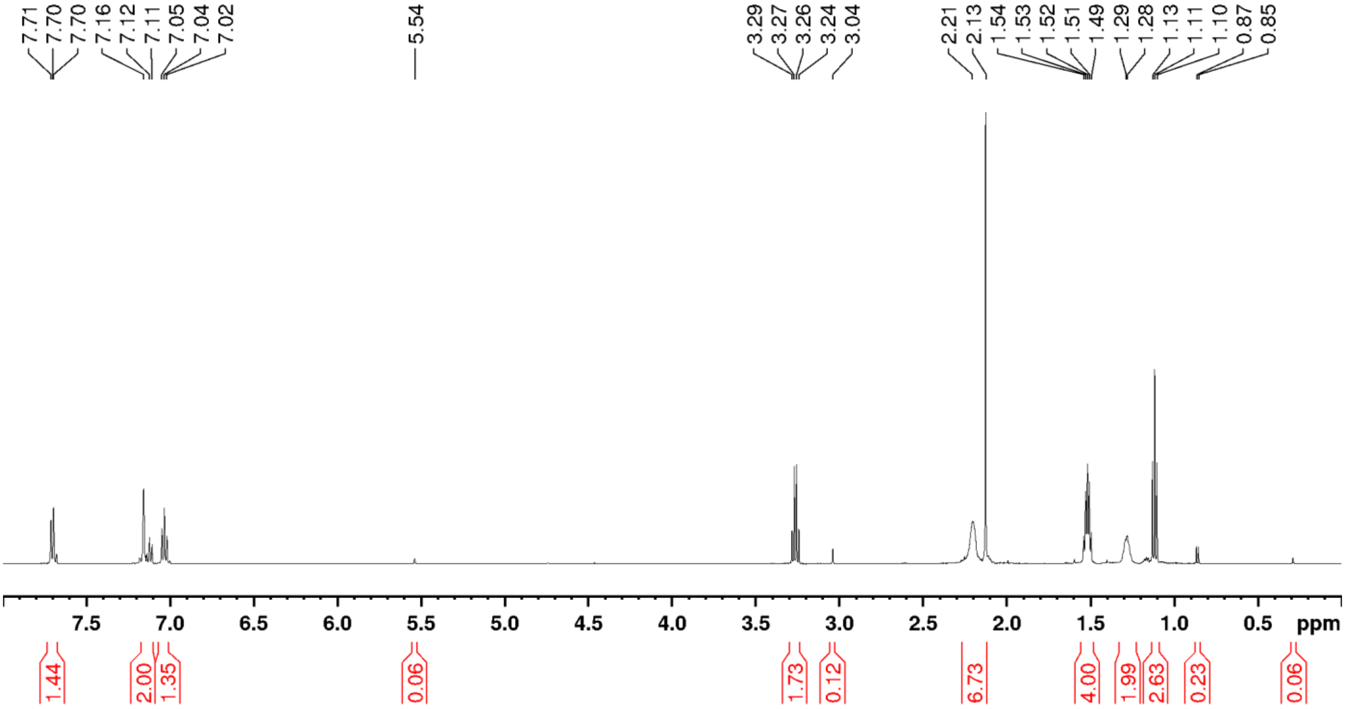

Figure S47: ^{1}H-NMR spectrum of reaction N24.

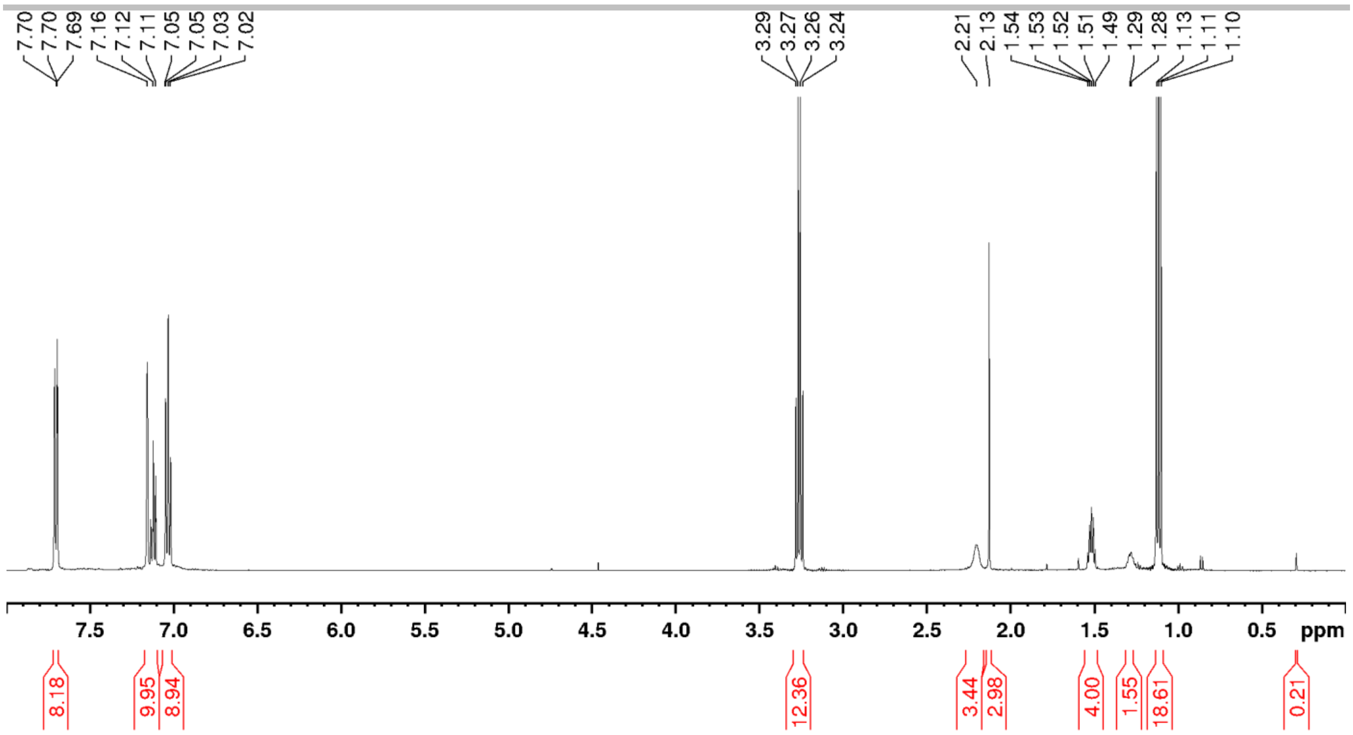

Figure S48: ^{1}H-NMR spectrum of reaction N25.

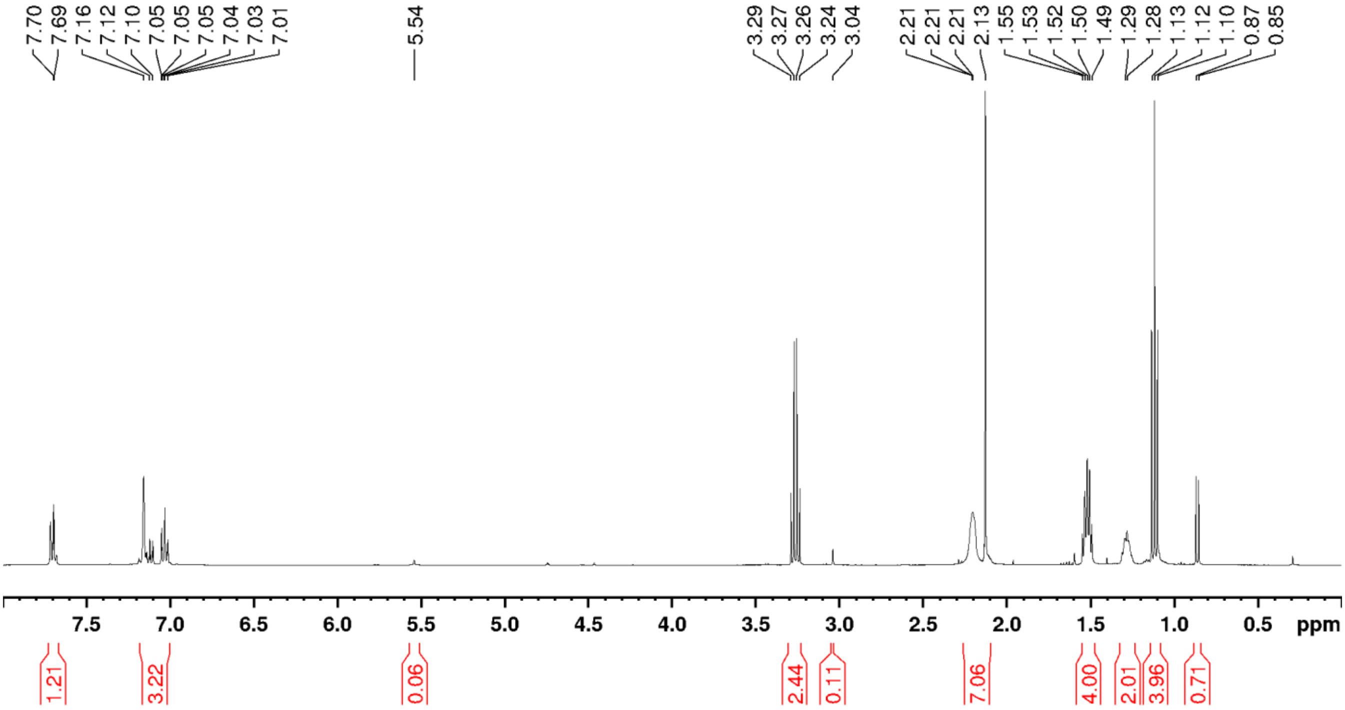

Figure S49: ^{1}H-NMR spectrum of reaction N26.

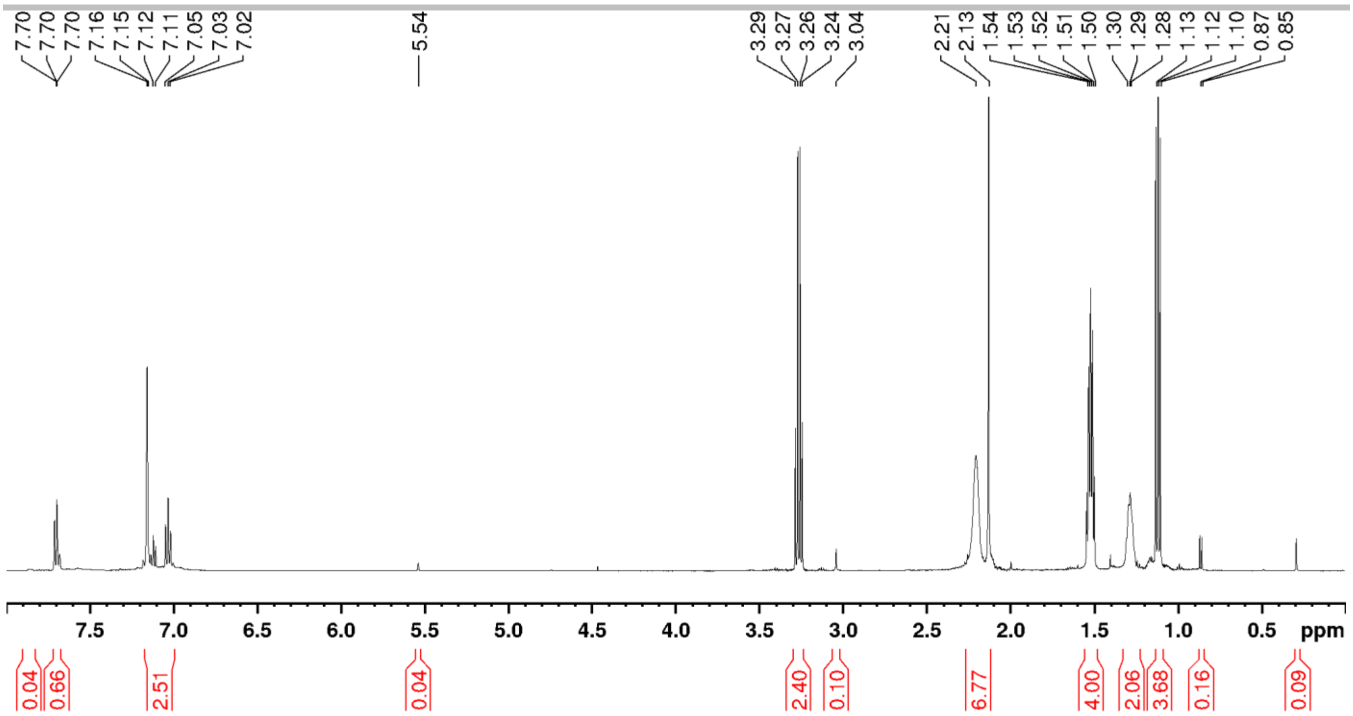

Figure S50: ^{1}H-NMR spectrum of reaction N27.

3.2.1 Conventional heating in a closed microwave vial

To compare the microwave radiation with conventional heating, we tested the best reactions conditions from the DoE experiment in a closed microwave vial under conventional heating in an oil bath.

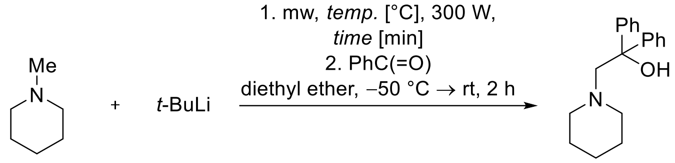

**1**	**3**	**7**
$C_6H_{13}N$	C_4H_9Li	$C_{19}H_{23}NO$
99.10 g·mol^{-1}	64.09 g·mol^{-1}	281.18 g·mol^{-1}

Tert-butyllithium (**3**, 1.00 eq., 4.00 mmol, 2.10 mL; 1.9 M in *n*-pentane) was added to an evacuated Schlenk flask and the contained *n*-pentane was removed via vacuum. Then *N*-methylpiperidine (**1**, 5.00 eq., 20.00 mmol, 2.45 mL) was added and the reaction solution was transferred to a microwave vial. The microwave vial was closed and the reaction solution was heated by conventional methods in an oil bath (100 °C, 120 min). The reaction solution was then cooled to –50 °C and benzophenone (1.20 eq., 4.80 mmol, 2.4 M in diethyl ether, 2.00 mL) was added. After stirring for 2 h at room temperature an NMR sample was taken.

As we saw from the DoE optimization, that the exact temperature is not essential for a successful deprotonation, we expected also temperature constancy in the microwave in comparison to conventional heating to be less relevant. Therefore, the resulting yield from both methods, determined by NMR spectroscopy, is comparable (52% conventional heating vs. 56% microwave heating). However, we do not want to recommend heating lithium alkyls in a closed vessel without pressure control. Explosion of the closed vessel and following uncontrolled exposure of hot lithium alkyls with moisture and air displays a safety risk. In the microwave however, the pressure stamp controls the reaction and measures the pressure *in situ*. In case of a very high pressure, the device stops heating without the risk of explosion of the vessel. In our DoE reaction optimization, we found that the closed setup is crucial for a successful reaction and we think that the microwave setup is one of the easiest and most comfortable ways to carry out such heating of lithium alkyls under pressure.

Additionally, further advantages of microwave heating, like temperature constancy, faster heating and controlled temperature gradient in the microwave may be important for further reaction systems with lithium alkyls that expand the first application of microwave heating for lithium alkyls that we present here.

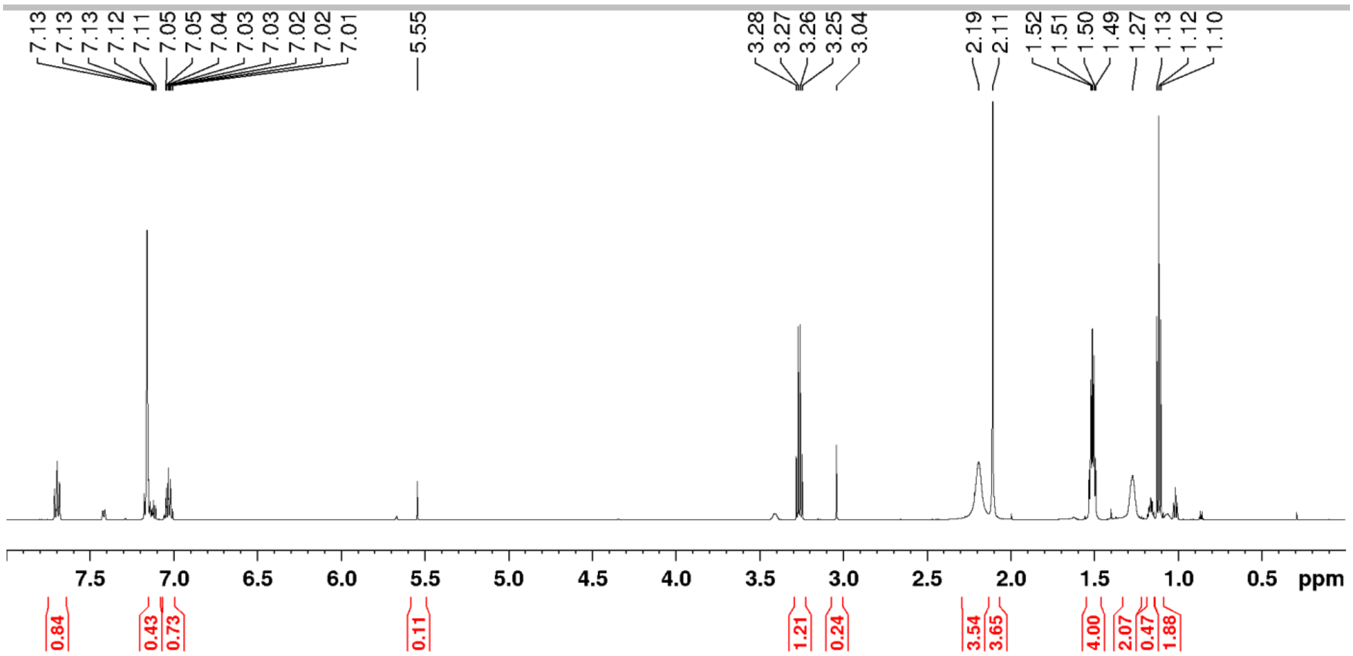

Figure S51: ^{1}H-NMR spectrum of deprotonation of *N*-methylpiperidine under conventional heating with best reactions conditions from DoE experiment.

References

[1] T. Kottke, D. Stalke, *J. Appl. Cryst.* **1993**, *26*, 615–619.
[2] Bruker, *Apex3*, Bruker AXS Inc., Madison, Wisconsin, USA **2018**.
[3] Bruker, *Apex4*, Bruker AXS Inc., Madison, Wisconsin, USA **2021**.
[4] O. V. Dolomanov, L. J. Bourhis, R. J. Gildea, J. A. K. Howard, H. Puschmann, *J. Appl. Cryst.* **2009**, *42*, 339–341.
[5] G. M. Sheldrick, *Acta Cryst.* **2015**, *A71*, 3–8.
[6] G. M. Sheldrick, *Acta Cryst.* **2015**, *C71*, 3–8.
[7] R. D. Dennington, T. A. Keith, J. M. Millam, *GaussView 6.0.*, Semichem Inc., Shawnee Mission, KS, USA **2016**.
[8] M. J. Frisch, G. W. Trucks, H. B. Schlegel, G. E. Scuseria, M. A. Robb, J. R. Cheeseman, G. Scalmani, V. Barone, G. A. Petersson, H. Nakatsuji, X. Li, M. Caricato, A. Marenich, J. Bloino, B. G. Janesko, R. Gomperts, B. Mennucci, H. P. Hratchian, J. V. Ortiz, A. F. Izmaylov, J. L. Sonnenberg, D. Williams-Young, F. Ding, F. Lipparini, F. Egidi, J. Goings, B. Peng, A. Petrone, T. Henderson, D. Ranasinghe, V. G. Zakrzewski, J. Gao, N. Rega, G. Zheng, W. Liang, M. Hada, M. Ehara, K. Toyota, R. Fukuda, J. Hasegawa, M. Ishida, T. Nakajima, Y. Honda, O. Kitao, H. Nakai, T. Vreven, K. Throssell, J. A. Montgomery, Jr., J. E. Peralta, F. Ogliaro, M. Bearpark, J. J. Heyd, E. Brothers, K. N. Kudin, V. N. Staroverov, T. Keith, R. Kobayashi, J. Normand, K. Raghavachari, A. Rendell, J. C. Burant, S. S. Iyengar, J. Tomasi, M. Cossi, J. M. Millam, M. Klene, C. Adamo, R. Cammi, J. W. Ochterski, R. L. Martin, K. Morokuma, O. Farkas, J. B. Foresman, and D. J. Fox, *Gaussian 09, Revision E.01*, Gaussian, Inc., Wallingford, CT, USA **2016**.
[9] S. Grimme, *WIREs Comput. Mol. Sci.* **2011**, *1*, 211–228.
[10] P. Flükiger, H. P. Lüthi, S. Portmann, J. Weber, *MOLEKEL 4.3*, Swiss Center for Scientific Computing, Manno, Switzerland **2000**.
[11] R.A. Finnegan, H. W. Kutta, *J. Org. Chem.* **1965**, *30*, 4138-4144.
[12] J. Kleinheider, C. Schwab, C. Strohmann, *Organometallics*, **2023**, *42*, 3173–3177.
[13] Bruker*, XP – Interactive molecular graphics. Version 5.1*, Bruker AXS Inc., Madison, Wisconsin, USA, **1998**.
[14] F. Becke, F. W. Heinemann, T. Rüffer, P. Wiegeleben, R. Boese, D. Bläser, D. Steinborn, *J. Organomet. Chem.* **1997**, *548*, 205–210.

Author Contributions

A.S.: experimental work for DoE, investigation, analysis of data for DoE, finalization of manuscript and SI, validation
R.S.: experimental work for structures, FTIR- and initial experiments, DFT-calculation, investigation, analysis of data, writing of first draft
A.O.: experimental work for DoE
L.B.: measurement of solid-state structure **5** and **8**
C.S.: funding acquisition, analysis of data, investigation, project administration
